# Refinement of the Diatom Episome Maintenance Sequence and Improvement of Conjugation-Based DNA Delivery Methods

**DOI:** 10.3389/fbioe.2016.00065

**Published:** 2016-08-08

**Authors:** Rachel E. Diner, Vincent A. Bielinski, Christopher L. Dupont, Andrew E. Allen, Philip D. Weyman

**Affiliations:** ^1^Microbial and Environmental Genomics Group, J. Craig Venter Institute, La Jolla, CA, USA; ^2^Integrative Oceanography Division, Scripps Institution of Oceanography, University of California San Diego, La Jolla, CA, USA; ^3^Synthetic Biology and Bioenergy Group, J. Craig Venter Institute, La Jolla, CA, USA

**Keywords:** diatom, bacteria, conjugation, genetic tools, episome, DNA delivery, DNA replication, *Phaeodactylum*

## Abstract

Conjugation of episomal plasmids from bacteria to diatoms advances diatom genetic manipulation by simplifying transgene delivery and providing a stable and consistent gene expression platform. To reach its full potential, this nascent technology requires new optimized expression vectors and a deeper understanding of episome maintenance. Here, we present the development of an additional diatom vector (pPtPBR1), based on the parent plasmid pBR322, to add a plasmid maintained at medium copy number in *Escherichia coli* to the diatom genetic toolkit. Using this new vector, we evaluated the contribution of individual yeast DNA elements comprising the 1.4-kb tripartite *CEN6-ARSH4-HIS3* sequence that enables episome maintenance in *Phaeodactylum tricornutum*. While various combinations of these individual elements enable efficient conjugation and high exconjugant yield in *P. tricornutum*, individual elements alone do not. Conjugation of episomes containing *CEN6-ARSH4* and a small sequence from the low GC content 3′ end of *HIS3* produced the highest number of diatom exconjugant colonies, resulting in a smaller and more efficient vector design. Our findings suggest that the *CEN6* and *ARSH4* sequences function differently in yeast and diatoms, and that low GC content regions of greater than ~500 bp are a potential indicator of a functional diatom episome maintenance sequence. Additionally, we have developed improvements to the conjugation protocol including a high-throughput option utilizing 12-well plates and plating methods that improve exconjugant yield and reduce time and materials required for the conjugation protocol. The data presented offer additional information regarding the mechanism by which the yeast-derived sequence enables diatom episome maintenance and demonstrate options for flexible vector design.

## Introduction

Diatoms play a critical role in marine ecosystems and global carbon cycling. They are also excellent candidates for bioproduction of valuable commercial compounds and renewable energy resources, as they display rapid growth rates across a range of environmental conditions. However, the development of diatom molecular tools to enhance understanding and enable genetic manipulation of diatom cellular capabilities is nascent and lags far behind molecular tool development in other prominent model species such as *Escherichia coli* (bacteria) and *Saccharomyces cerevisiae* (yeast). The diatom *Phaeodactylum tricornutum* has emerged as an important model for examining diatom biological processes and commercial potential and as a test strain to develop new genetic tools that can be expanded into other diatom species (Apt et al., 1996; Lopez et al., 2005; Siaut et al., 2007; Bozarth et al., 2009). *P. tricornutum* was the first diatom to be genetically transformed *via* biolistic particle delivery (Apt et al., 1996) and the first to be genetically modified using RNAi-mediated gene silencing (De Riso et al., 2009). Complete gene knockout mutations are also possible using TALEN and CRISPR/CAS9 technology (Daboussi et al., 2014; Weyman et al., 2014; Nymark et al., 2016). Recently, *P. tricornutum* was shown to stably maintain engineered diatom episomes delivered to the diatoms *via* bacterial conjugation, which represents a major advancement in diatom tool development but requires further optimization (Karas et al., 2015).

Diatom episomes potentially allow for consistent and predictable protein expression, perhaps for entire biochemical pathways. Conjugation-based episome delivery may facilitate simplified delivery of genome editing components (e.g., CRISPR/CAS9), allow complementation of genetic mutants in reverse genetics approaches, and provide a platform for overexpressing proteins of interest for functional or subcellular localization studies. Episomes avoid complications associated with random integration of transgenic DNA into diatom nuclear chromosomes, such as multiple or partial expression cassette insertions, interruption of non-target genes, and insertion position expression effects (Dunahay et al., 1995; Falciatore et al., 1999; Miyagawa et al., 2009). Episomes can be introduced into diatoms *via* bacterial conjugation, which is the most efficient method of transgene delivery established to date. Episomes can also be delivered by biolistic particle delivery (confirmed in our lab, data not shown), electroportation, and PEG-mediated transformation (Karas et al., 2015). The multiple methods of episome delivery make them a versatile addition to the diatom molecular toolkit. Our conjugation protocol (Table S1 in Supplementary Material) employs a two-plasmid system in which a mobilizable “cargo plasmid” is transferred to the diatom cell by a conjugative plasmid (pTA-MOB) that itself cannot be mobilized due to deletion of the origin of transfer (*oriT*) (Strand et al., 2014). Diatom episomes must be capable of replication and segregation during division in both the donor bacteria (if conjugation is the preferred delivery method) and in the diatom recipient. In model species of bacteria and yeast, mechanisms of replication are well understood (Murray and Szostak, 1983; Del Solar et al., 1998) and can be varied to accommodate a range of experimental goals. In diatom recipients, the only genetically tested examples of episome maintenance rely on a foreign 1.4-kb DNA sequence derived from yeast, which permits maintenance in both centric and pennate diatoms (Karas et al., 2015). However, very little is known about how the foreign DNA sequence enables maintenance in diatoms.

The DNA sequence *CEN6-ARSH4-HIS3* (CAH), originally derived from a yeast artificial chromosome, consists of three contiguous individual elements, which function in yeast as a centromere (*CEN6*), an origin of replication (*ARSH4*), and a selectable marker (*HIS3*). The *CEN6* sequence, we used in this study, is a 117-bp region of very low GC content (14%); in yeast, this sequence is sufficient for complete centromere function during both meiosis and mitosis (Newlon, 1988; Cottarel et al., 1989). *ARSH4* is a well characterized yeast replication origin enabling initiation of DNA replication (Stinchcomb et al., 1980). Replication origins [also called autonomously replicating sequences (Ars)] are typically found throughout eukaryotic genomes, and the yeast Ars contains a small consensus sequence (Newlon and Theis, 1993; Wyrick et al., 2001; Nieduszynski et al., 2006; Siow et al., 2012; Tagwerker et al., 2012). Only one Ars is required for maintenance of smaller plasmids, while larger plasmids (e.g., greater than 160–300 kb) are more easily assembled and maintained using multiple Ars sequences (Muller et al., 2012; Karas et al., 2013). *ARSH4* is a 388-bp region of low GC content (32%). In addition to the *CEN6* and the *ARSH4*, the final component is a selectable marker that provides a gene to complement yeast histidine (His) auxotrophy, often used for positive selection of successful transformants (Weinstock and Strathern, 1993). The *HIS3* sequence of 872-bp is 45% GC content, which is slightly lower than the average 47% GC content of the *P. tricornutum* genome (Weinstock and Strathern, 1993; Bowler et al., 2008). The CAH region was initially included in diatom episomes to enable yeast-based plasmid assembly methods and was later found to be the element responsible for diatom episome maintenance (Karas et al., 2015). It was unclear, which of these elements individually or in combination were essential for diatom plasmid maintenance, or, given the role of the CAH in supporting stable diatom episome maintenance, whether these elements performed similar functional roles in diatoms as in yeast.

In this study, we explored the sequence requirements for diatom episome maintenance and developed new molecular tools and protocols to facilitate the transfer of episomes from *E. coli* to the diatom *P. tricornutum*. Our objectives were threefold: (1) develop a new diatom episome with medium copy number in *E. coli* for stable and effective genetic engineering, (2) identify elements and characteristics of the CAH sequence that enable diatom episome maintenance, and (3) improve upon the current methods for bacterial transfer of DNA to diatoms. To accomplish these goals, we engineered a cargo vector based on the plasmid pBR322 (Sutcliffe, 1979) that can be efficiently transferred into and maintained in *P. tricornutum*. Using this vector, we identified sub-sequences from CAH that were sufficient for episome maintenance, which permitted new insights into understanding plasmid maintenance requirements and construction of a smaller cargo vector. Finally, we developed modifications of the conjugation protocol, which boost efficiency while saving time and materials and are compatible with high-throughput methods for multiple conjugations.

## Materials and Methods

### Strains and Growth Conditions

The diatom *P. tricornutum* strain CCMP 632 was obtained from the Provasoli–Guillard National Center for Culture of Marine Algae and Microbiota (NCMA) and was cultured in filter-sterilized (0.2 μm bottle-top filters, Thermo Fisher Scientific, Waltham, MA, USA) L1 artificial seawater medium with Aquil as a base (Price et al., 1989) as described by NCMA (http://ncma.bigelow.org/) (Table S2 in Supplementary Material). Plates for culturing the diatoms on solid medium consisted of 50% full strength L1 medium and 50% melted 2% agar in milli-Q water (autoclave sterilized), both brought to a temperature of 50°C separately prior to mixing. The resulting plates are 1/2 × L1 and 1% final agar concentration (1/2 × L1-agar plates). Cultures were maintained at 18°C with a 60 μmol photons m^−2^ s^−1^ light intensity, and a 14/10-h light/dark cycle. Diatom cultures were xenic, however, bacterial numbers were kept low by regular maintenance with antibiotics. Diatom cell numbers were counted using disposable improved-neubauer haemocytometers (IN-Cyto, Chungnam-do, Korea). Antibiotics for selection of diatom exconjugants were provided as 20 μg mL^−1^ Phleomycin (Phleo) on agar plates or Zeocin (50 μg mL^−1^) in liquid cultures.

*Escherichia coli* TransforMax EPI300 cells (Epicenter, Madison, WI, USA) were used for plasmid cloning and conjugative transfer of plasmids to diatoms (Table S2 in Supplementary Material). Cultures were grown in LB broth (Amresco, Solon, OH, USA) at 37°C in a shaking (225 rpm) incubator. Antibiotics as needed were provided as 20 μg mL^−1^ gentamicin (Gm) in water solvent, 100 μg mL^−1^ ampicillin (Amp), 50 μg mL^−1^ kanamycin (Kan), 5 μg mL^−1^ tetracycline (Tet) in ethanol solvent, 10 μg mL^−1^ chloramphenicol (Cm) in ethanol solvent.

### Plasmid Design and Construction

All plasmids were constructed by Gibson Assembly of amplified PCR products (Gibson et al., 2009). Templates and primers used for all plasmid assemblies are listed in Tables S3,S4 in Supplementary Material. PCR products were amplified using PrimeStar Max DNA polymerase mastermix (Takara Clontech, Mountain View, CA, USA) using the manufacturer’s recommended protocol and were confirmed by agarose gel electrophoresis. PCR products were purified using Qiagen PCR purification kits (Qiagen, Hilden, Germany) and quantified prior to Gibson assembly using a Nanodrop Spectrophotometer (Thermo Fisher Scientific, Waltham, MA, USA). Assembled plasmids were transformed into *E. coli* by electroporation using 1 mm cuvettes and the preset bacterial transformation protocol (Bio-Rad Laboratories, Irvine, CA, USA). Individual colonies were isolated by selective plating on LB + Amp + Tet. PCR screening of colonies was performed using the primers ptrepinsertscrn1 and ptrepinsertscrn2 (Table S3 in Supplementary Material) followed by plasmid purification using the Qiagen miniprep kit (Qiagen, Hilden, Germany) and various diagnostic restriction enzyme digests (New England Biolabs, Ipswich, MA, USA) to verify plasmid construction.

**Figure 1:**
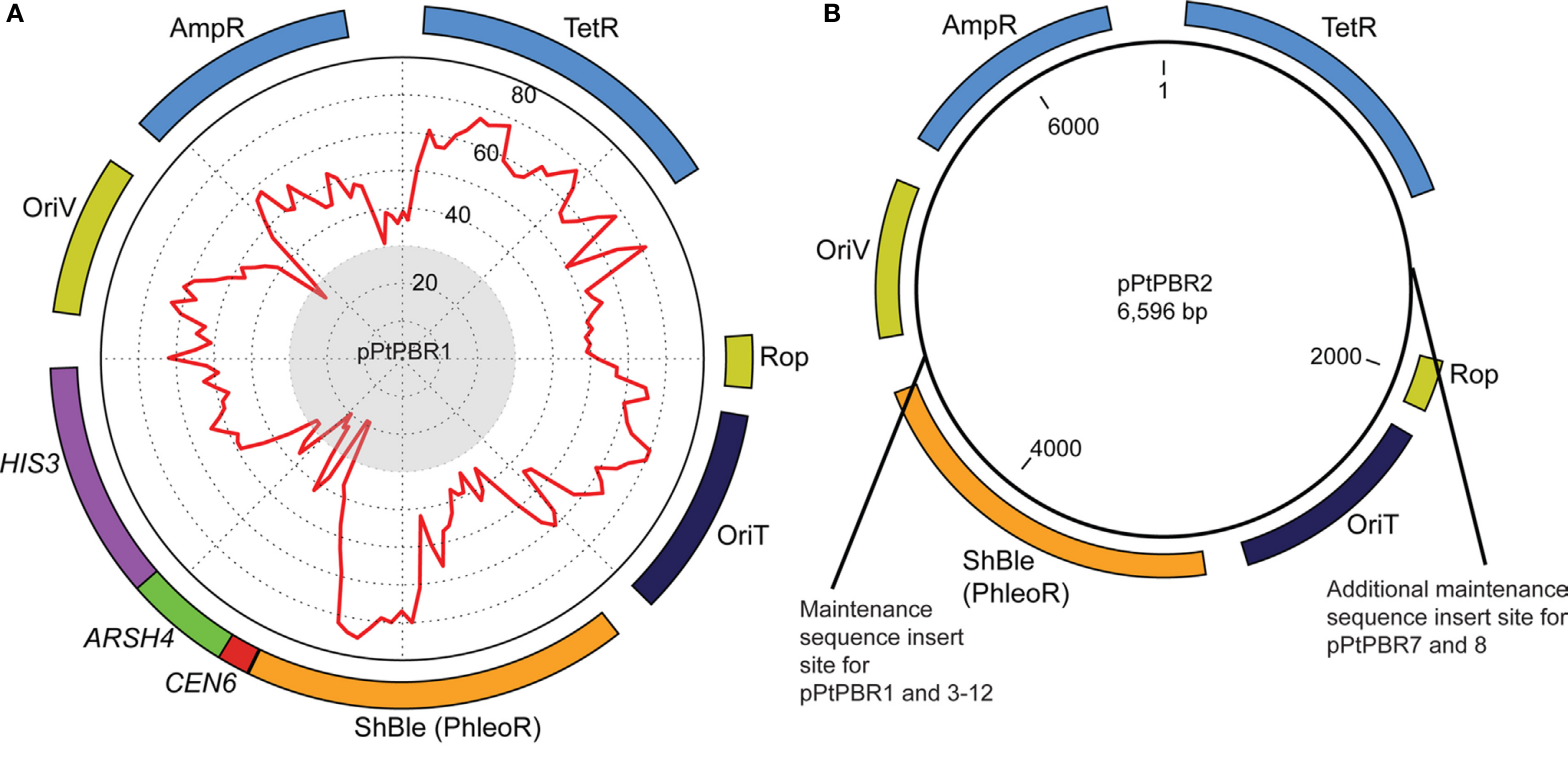
**Plasmid maps of (A) pPtPBR1 and (B) pPtPBR2, which both contain Ampicillin (AmpR) and Tetracyline (TetR) resistance cassettes for *E. coli* selection, a diatom ShBle cassette for diatom selection on phleomycin (PhleoR), an origin of transfer enabling conjugative transfer of episomes from bacteria into diatoms (derived from the plasmid pRL2948a), and an origin of replication (OriV) and the rop gene, which reduces plasmid copy number**. The plasmid pPtPBR1 **(A)** contains the *CEN6-ARSH4*-*HIS3* episome maintenance sequence. Percent GC content for each region of the plasmid is graphically displayed as a red line on a graph inside the plasmid and was constructed using a 100-bp window advancing every 50-bp. Regions with a GC content of less than 30% fall within the gray central circle of the graphic. The pPtPBR2 plasmid **(B)** contains no CAH region. The pPtPBR3-12 plasmids share a common backbone with pPtPBR2 with additional modifications indicated in the **(B)**.

We used the plasmid backbone from pBR322 for all plasmids tested in this study. Plasmid pBR322 is derived from a natural variant of the ColE1 plasmid called MB1 (Bolivar et al., 1977) and contains an *oriT* [originally called basis of mobilization (*bom*)] that is compatible with RP4-mediated conjugation systems (Finnegan and Sherratt, 1982) (Table S2 in Supplementary Material). We replaced the pBR322 *oriT* with the *oriT* from RP4 to ensure maximum transfer efficiency, as this *oriT* has been shown to enable conjugation into diatoms previously (Karas et al., 2015). Two base plasmids were constructed to serve as positive and negative controls for further experiments: pPtPBR1, which contains the CAH sequence (Figure 1A), and pPtPBR2, which lacks CAH (Figure 1B). To construct pPtPBR1, the pBRR322 backbone lacking the pBR322 *oriT* was combined with an insert containing the RP4 *oriT* and the ShBle cassette amplified from pPtPuc3-Km using primers Insert-F and Insert-R (Table S3 in Supplementary Material). To construct pPtPBR2, the pBRR322 backbone lacking the pBR322 *oriT* was combined with an insert containing the RP4 *oriT* and the ShBle cassette using primers Insert-F and Insert-R (Table S3 in Supplementary Material).

The plasmids pPtPBR3 through pPtPBR12 were constructed by two-piece Gibson assembly of a vector PCR product and an insert PCR product. Plasmid sources for both vector and insert products, as well as primers used, are listed in Table S4 in Supplementary Material. Plasmids pPtPBR3 (*CEN6* only), pPtPBR4 (*ARSH4* only), pPtPBR5 (*HIS3* only), and pPtPBR6 (*CEN6*-*ARSH4*) were constructed using previously assembled and verified pUC-based versions already containing the specified portions of the CAH region as templates (Table S4 in Supplementary Material). Inserts containing the RP4 *oriT*, the ShBle cassette, and the specified portion of the CAH sequence were then amplified by PCR using the Insert-F and Insert-R primers. These inserts were combined with the pBR322 backbone vector with the *oriT* site omitted as described in the construction of pPtPBR1 and pPtPBR2 above, using primers pBR322-F and pBR322-R.

The remaining plasmids were constructed using plasmids constructed above as templates (Table S4 in Supplementary Material). Plasmid pPtPBR7 has both *CEN6* and ARS4 elements, but, in contrast to pPtPBR6, has these elements located on different parts of the plasmid. The plasmid pPtPBR7 was constructed using pPtPBR3, containing only *CEN6*, as a backbone template and pPtPBR1 as an insert template to amplify the ARS4, which was inserted between the tetracycline resistance cassette (TetR) and the *Rop* gene (Figure 1B). A similar procedure was used to construct pTPBR8, which contains two sets of *CEN6*-ARS4 regions on separate sides of the plasmid in the same location as the pPtPBR7 elements (Figure 1B). For this, pPtPBR6 was used for both a template and insert (Table S4 in Supplementary Material). Plasmids pPtPBR9 through pPtPBR12 were constructed by amplifying the pPtPBR1 backbone using Gibson assembly primers, omitting particular regions. Plasmid pPtPBR9 contains only the ARS4 and *HIS3* sequences. Plasmid pPtPBR10, 11, and 12 contain the *CEN6* and ARS4 elements along with portions of the beginning of the *HIS3* sequence (100, 200, and 300 bp, respectively).

### Plasmid Sequencing and Deposit

The plasmids pPtPBR1, pPtPBR2, and pPtPBR11 constructed in this study were fully sequenced (Eurofins Genomics, Louisville, KY, USA). Primers for all sequencing and PCR amplifications were obtained from Integrated DNA Technologies (Coralville, IA, USA) and are listed in Table S3 in Supplementary Material. Sequences for the plasmids are available in Table S7 in Supplementary Material and sequences as well as annotations are deposited in NCBI Genbank with the following accession numbers: KX523201 (pPtPBR1), KX523202 (pPtPBR2), and KX523203 (pPtPBR11). Plasmids were also deposited with AddGene.

### Conjugation of Episomes into Diatoms and Plasmid Rescue

We used the conjugation protocol described in Karas et al. (2015) and also tested various plating and culturing modifications in an effort to improve the conjugation protocol for various applications. A detailed protocol of the original method and modifications presented in this study are presented in Table S1 in Supplementary Material. To examine the sequence elements required to produce an optimal number of exconjugants, conjugations were conducted in triplicate using the previously established protocol (Karas et al., 2015). Pre-plated diatom cultures grown on 1/2 × L1-agar plates were resuspended in L1 medium and adjusted to a final cell concentration of about 5.5 × 10^8^ cells mL^−1^. Overnight *E. coli* cultures containing the RP4-derived conjugative plasmid pTA-MOB (Strand et al., 2014), which contains no *oriT*, and the desired cargo plasmid/episome for delivery into the diatoms were diluted 1:50 into fresh medium containing appropriate antibiotics and grown to an OD_600_ of 0.8–1.0. Cultures (50 mL) were then centrifuged for 10 min at 3500*g*, supernatant was removed, and the *E. coli* cells were resuspended in 600 μL SOC medium. 200 μL each of *E. coli* and *P. tricornutum* concentrated culture were mixed, plated on 1/2 × L1-agar plates containing 5% LB medium (made with an L1 seawater base) and incubated at 30°C in the dark for 90 min. Plates were then moved to typical diatom culturing conditions and allowed to recover for 2 days, prior to resuspending cells in 1.5 mL of L1 medium and plating a portion of the reaction on selective 1/2 × L1-agar plates supplemented with Phleo for exconjugant selection. To confirm the presence of episomes, DNA was extracted from the exconjugant diatoms using the protocol described in Karas et al. (2015) and then transformed into *E. coli* using electroporation to effectively “rescue” replicating plasmids (Karas et al., 2015). If DNA was maintained as an episome in the diatom and later transformed into *E. coli*, it would subsequently replicate in *E. coli* as well since the necessary elements for maintenance are present on the plasmid. Alternatively, if selection in diatom exconjugants is not due to maintanence of an episome, *E. coli* colonies will not be able to be recovered after transformation of exconjugant DNA. To ensure absence of *E. coli* carry-over contamination, colonies were passaged multiple times on 1/2 × L1-agar plates and were also patched onto LB agar plates and incubated overnight at 37°C; no *E. coli* growth was observed.

For optimization experiments, various modifications were made to the conjugation protocol. The cargo plasmid pPtPBR11 was used in optimization experiments, as this plasmid had high exconjugant yield and a smaller size than pPtPBR1 (see Section “Results” and “Discussion”). For development of a high-throughput option, 12-well Costar (Corning, Corning, NY, USA) plates were utilized, either in lieu of or in conjunction with standard 100 mm Petri dishes (Denville Scientific, South Plainfield, NJ, USA). We first scaled down the Karas et al. (2015) protocol reaction volume by roughly fivefold. Melted 1/2 × L1-agar-5% LB (~3.5 mL) was pipetted into each well of a 12-well cell culture plate and allowed polymerize (Table S1 in Supplementary Material). Liquid *P. tricornutum* cultures were harvested by centrifugation for 10 min at 3,000*g* (room temperature) and adjusted to 5 × 10^8^ cells mL^−1^. 50 μL of the cell suspension was pipetted into the center of each agar “well” and lightly spread around with a loop or by rotating the plate to cover an area covering about 80% of the agar, leaving a gap between the cell suspension and the well edges. Plates were then dried in a laminar flow hood until no visible liquid remained and, subsequently, incubated for 96 h in the standard diatom culturing conditions (see above). On the day of conjugation, an overnight culture of *E. coli*-containing plasmids pPtPBR11 and pTA-MOB was diluted 1:50 in fresh liquid LB medium with antibiotics (10 μg mL^−1^ Tet, 100 μg mL^−1^ Amp, and 20 μg mL^−1^ Gent) incubated at 37^o^C until reaching an OD_600_ of 0.8. We, then, harvested bacterial cells by centrifugation (10 min at 3,000*g*, room temperature) and concentrated 100-fold by resuspension in SOC medium. 50 μL of either SOC alone (negative conjugation control) or bacterial suspension was added on top of the dried algal culture and plates rotated by hand to allow the bacteria to cover the algal area. The plates were dried in a laminar flow hood for ~10 min until no visible liquid remained and were covered and transferred to 30^°^C for 90 min (dark). They were then returned to typical diatom culturing conditions. After a 48-h recovery period, the cells in each well resuspended in 500 μL L1 liquid medium, and the entire reaction was replated on standard 100 mm Petri dishes containing 30 mL of 1/2 × L1-agar supplemented with 20 μg mL^−1^ Phleo. These plates were allowed to dry briefly in a laminar flow hood and then incubated for ~7–10 days in diatom culturing conditions until colonies were large enough to count.

To address the effect of pre-conjugation diatom plating conditions, we tested various reduced time intervals between plating and conjugation. Two 12-well 1/2 × L1-agar-5% LB agar plates were prepared as described above and plated with increasing number of cells on 4 successive days before conjugation, assuming a doubling time of ~1 doubling per day under these growth conditions. Therefore, on *t* = Day 4 (with *t* = Day 0 being the day of conjugation) cells were harvested from liquid culture and adjusted to 1 × 10^8^ cells mL^−1^ in L1 medium, and a 50 μL aliquot of this suspension (5 × 10^6^ cells) was plated onto each of 6 wells (3 for pPtPBR11 and 3 SOC negative controls). On *t* = Day 3, a total of 1 × 10^7^ cells were plated onto each of an additional 6 wells, followed by 2 × 10^7^ cells on *t* = Day 2 and 4 × 10^7^ cells on *t* = Day 1. Plate cultures were grown under standard diatom growth conditions. On *t* = Day 0, the overnight *E. coli* pPtPBR11/pTA-MOB culture was diluted (1:50) in fresh LB and grown to 0.8 OD_600_ with shaking at 37^°^C. We concentrated 25 mL by centrifugation (as above) and resuspended in 250 μL SOC medium. 50 μL of this suspension was added to each of the experimental treatment wells containing diatom cultures, and 50 μL of sterile SOC medium was added to the remaining wells (negative controls). After drying, these conjugations were carried out as described above with a 48-h recovery period followed by replating onto 100 mm 1/2 × L1-agar plates supplemented with 20 μg mL^−1^ Phleo. Diatom colonies were counted after ~10 days.

We also tested recovery time required before plating diatom conjugation reactions onto selective medium. For this experiment, 12 wells of 1/2 × L1-agar-5% LB were plated with 4 × 10^7^ cells on the day before conjugation and grown overnight in diatom culturing conditions. On the day of the conjugation, *E coli* pPtPBR11/pTA-MOB was cultured and prepared as described above. 50 μL of SOC was added to 3 wells, while 50 μL of bacterial suspension was added on top of the other 9 wells. The conjugation plate was incubated at 30^o^C for 90 min, after which 4 wells (1 SOC control, 3 pPtPBR11/pTA-MOB) were immediately resuspended into 500 μL L1 liquid medium, while the plate with the remaining reactions was returned to diatom growth conditions for recovery. The four cell suspensions were then plated on 100 mm 1/2 × L1-agar plates supplemented with 20 μg mL^−1^ Phleo and incubated at 18^o^C after drying (*t* = Day 0 treatment). The next day (24 h after the incubation at 30°C for 90 min), 4 more wells (1 SOC control, 3 pPtPBR11/pTA-MOB) were resuspended and replated on selective medium (*t* = Day + 1), and the final four wells resuspended and replated on *t* = Day + 2 (48 h after the incubation at 30°C for 90 min). The final day 2 plating was similar to the optimized protocol previously described and thus served as a positive control (Karas et al., 2015). Selection plates were incubated for 7–10 days in diatom culturing conditions, after which colonies were counted.

### Plasmid Maintenance

Three plasmids (pPtPBR1, 6, and 8) were tested for maintenance following the protocol in Karas et al. (2015). Following conjugation and selection on Phleo 1/2 × L1-agar plates, 2 colonies of pPtPBR1, 2 colonies of pPtPB8, and 1 colony of pPtPBR6 were transferred to liquid L1 medium containing no antibiotics. Cultures were transferred to fresh medium weekly for 30 days and then plated on non-selective 1/2 × L1-agar plates to obtain single colonies (Figure S1A in Supplementary Material). Between 45 and 100 colonies from each treatment were patched onto non-selective 1/2 × L1-agar plates, and, after about 1 week of growth, each patch was repatched onto both non-selective medium as a positive control and selective medium to determine how many retained the transgene or, minimally, the antibiotic resistance cassette (Figures S1A,B in Supplementary Material).

## Results

### Sequence Elements Enabling Efficient Plasmid Maintenance in *P. tricornutum*

**Figure 2:**
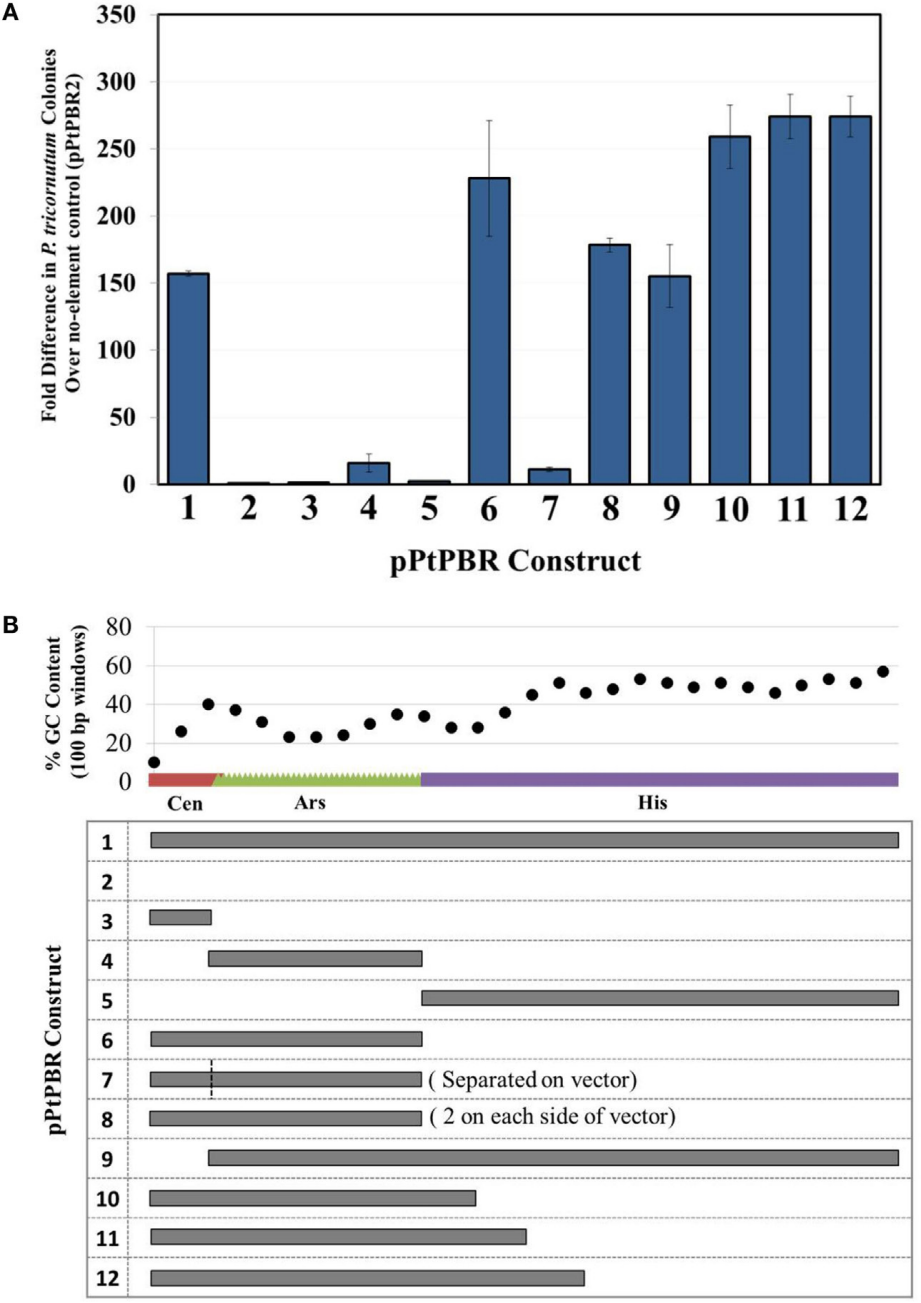
**(A)** Diatom colony yields, presented as fold difference from the negative control plasmid pPtPBR2 (no maintenance elements), for plasmids containing various potential maintenance sequences. *N* = 3, error bars = SD, **(B)** relative size and GC content analysis of possible maintenance sequences contained in plasmids pPtPBR1-12.

We aimed to determine which combinations of sub-sequences within the yeast *CEN6-ARSH4-HIS3* were sufficient to allow episome maintenance. We found that the newly engineered plasmid pPtPBR1, containing the full CAH sequence, resulted in greater than 150-fold more diatom exconjugants than the negative control plasmid pPtPBR2 lacking the CAH sequence (Figure 2A). Plasmids from pPtPBR1 were effectively rescued in *E. coli* (Table S5 in Supplementary Material), confirming their successful maintenance as episomes in diatoms. Furthermore, these plasmids were maintained in non-selective medium at levels similar to those previously reported in yeast and diatoms (Karas et al., 2015) (Figure S1B in Supplementary Material). Conjugation with pPtPBR2 yielded a very small number of diatom colonies that could not be recovered *via E. coli* rescue. Thus, pPtPBR1 and pPtPBR2 function similarly to the previously described plasmids containing or lacking the CAH sequence, respectively (Karas et al., 2015). In this study and in the previous study, we observed that conjugation of an episome containing the CAH sequence resulted in high diatom colony numbers. Additionally, these episomes were shown to be maintained in diatoms and could be rescued in *E. coli*. Thus, high diatom colony numbers resulting from a conjugation relative to the pPtPBR2 control suggest the successful transfer and maintenance of an episome.

When the individual CAH sequence elements were tested separately for episome maintenance ability, conjugation efficiency was very low. The numbers of diatom colonies emerging after conjugation with *E. coli* strains bearing episomes containing only *CEN6* or *HIS3* were similar to the no element control pPtPBR2 (Figure 2A). Episomes containing only the *ARSH4* sequence for maintenance resulted in a slightly higher number of diatom colonies, ~16-fold higher than the no element control, though 67-fold less than the pPtPBR1 positive control. When plasmid DNA was extracted from some of the few *P. tricornutum* lines obtained after conjugation with for *E. coli* bearing *CEN6*- or *HIS3*-only containing plasmids, no *E. coli* colonies were obtained, suggesting the absence of episomes in the diatoms and chromosomal integration of the marker. Episome rescue with DNA extracted from episomes containing only *ARSH4* resulted in a small number of *E. coli* colonies (Table S5 in Supplementary Material). Plasmids that contained the *CEN6*-*ARSH4* region without the *HIS3* (pPtPBR6) and the *ARSH4*-*HIS3* region without the *CEN6* (pPtPBR9) yielded a large number of *P. tricornutum* colonies, similar in number to the control plasmid pPtPBR1. One colony of pPtPBR6 was tested for maintenance during passage without selection for 30 divisions and was found to be maintained at levels similar to pPtPBR1 and to those previously reported (Karas et al., 2015) (Figure S1B in Supplementary Material). Plasmid pPtPBR7, which contained *CEN6* and *ARSH4* that were spatially separated by 3 kb (Figure 1B) resulted in a similar number of exconjugant *P. tricornutum* colonies as the pPtPBR4, containing the *ARSH4* only. Plasmid pPtPBR8, which contained two *CEN6*-*ARSH4* regions on different parts of the episome yielded a large a number of colonies similar to the pPtPBR1 positive control (Figure 2A) and the pPtPBR6 plasmid containing one copy of the *CEN6*-*ARSH4* region. Of the two pPtPBR8 colonies tested for plasmid maintenance during serial passage in the absence of selection, one was maintained stably while the other was maintained poorly compared to prior results (Figure 1B in Supplementary Material).

The 3′ region of the *HIS3* element contained a relatively high GC content compared to the first 200-bp of the 5′ region of *HIS3* adjacent to the *CEN6*-*ARSH4* sequence, which were low in GC content (Figure 2B). To examine whether this distinction plays a role in diatom episome maintenance, we tested variations of the CAH sequence with modified *HIS3* sequences. The plasmids pPtPBR10–12 contained the *CEN6*-*ARSH4* regions as well as some of the low GC content sequence adjacent to the *ARSH4* sequence but lacked the relatively high GC content portion of the *HIS3* sequence (Figure 2B). Each of these constructs produced similar numbers of diatom colonies to each other and more than the pPtPBR1 positive control containing the full CAH sequence (Figure 1B).

### Efficiency of Modified Conjugation Protocols

Culturing diatoms on 1/2 × L1-agar supplemented with 5% LB medium led to faster diatom growth and a higher number of exconjugant colonies after adjusting for diatom cell number (Figure S2 in Supplementary Material). After 4 days of growth on either 1/2 × L1-agar only or 1/2 × L1-agar-5% LB plates, diatom cell numbers were more than 2 times higher on LB supplemented plates: 1.59 × 10^8^ cells per plate on 1/2 × L1-agar and 3.59 × 10^8^ cells/plate when supplemented with 5% LB (Figure S2 in Supplementary Material). After resuspending diatom cells prior to conjugation, cell numbers were adjusted for each culture to 5 × 10^8^ cells mL^−1^. Following the standard conjugation protocol, after plating on selective medium, there were more than two times as many exconjugant colonies in the treatments where diatoms were supplemented with 5% LB agar (Figure S2 in Supplementary Material; Table S6 in SupplementaryMaterial). This experiment was repeated independently, also in triplicate, and similar patterns were observed (Table S6 in Supplementary Material).

**Figure 3:**
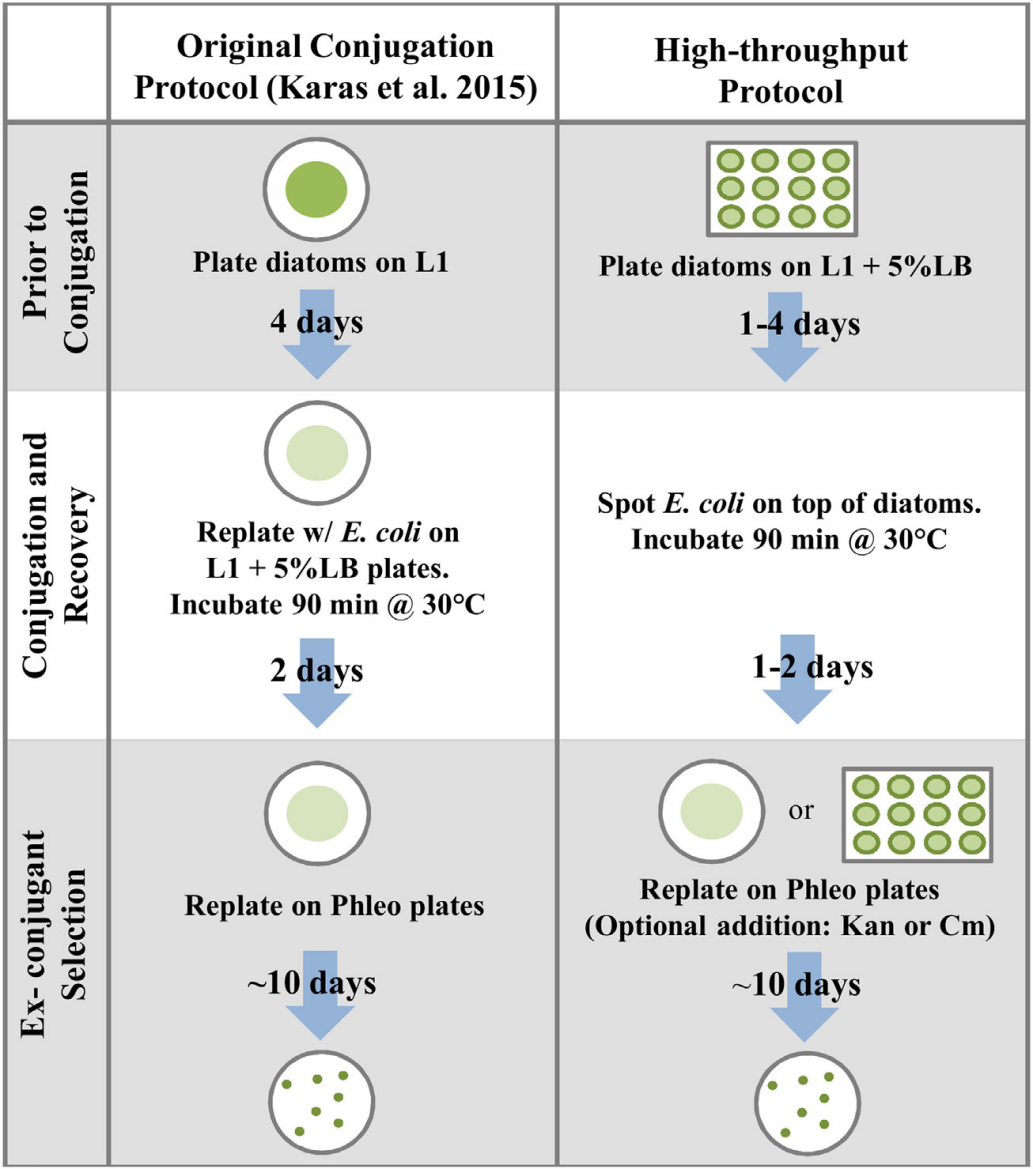
**Comparison of the protocol established in Karas et al. (2015) to the high-throughput conjugation protocol developed in this study**.

We developed a high-throughput method of conjugation based on plating diatoms on 5% LB medium in 12-well plates, followed by the direct addition of *E. coli* culture on top of the diatoms (Figure 3). Using the standard timing for diatom pre-plating and post-conjugation recovery (plated 4 days prior to conjugations and plated on selective medium 2 days after the 90-min incubation at 30°C), and after replating each entire conjugation reaction (for each of three replicates) on one 100 mm 1/2 × L1-agar plate containing Phleo 20 μg mL^−1^, an average of 146 diatom colonies were obtained per plate (Table S6 in Supplementary Material).

When testing recovery time after conjugation using the high-throughput protocol, plating cells immediately after the 30°C step (*t* = Day 0) resulted in only 5 colonies total (across 3 plates), whereas incubating conjugation reactions for 24 h resulted an average of 79 diatom colonies (Table S6 in Supplementary Material). Allowing for the full 48-h incubation before plating on selective medium resulted in a recovery of 3 times as many colonies, an average of 242 colonies, as the 24-h recovery time (Table S6 in Supplementary Material). There was little difference in the number of diatom exconjugants when the diatoms were plated at *t* = Day 4, 3, 2, or 1 prior to the conjugation (Table S6 in Supplementary Material). Furthermore, the number of exconjugant colonies was not affected when the recovery step was conducted on 1/2 × L1-agar plates with the antibiotics kanamycin or chloramphenicol in addition to phleomycin to ensure the *E. coli* culture was killed (Table S6 in Supplementary Material).

## Discussion

### The pPtPBR Plasmids: A New Series of Diatom Conjugation Vectors with Medium Copy Number in *E. coli*

For a robust genetic model system, a variety of plasmid vectors are often developed and optimized depending on the ultimate use, including efficient cloning of the gene(s) of interest in *E. coli*. Important factors to consider include cloning insert and vector size, ease of cloning, restriction sites, and plasmid copy number. Vectors based on bacterial artificial chromosomes (BACs), such as the p0521s plasmid used in Karas et al. (2015), contain a BAC backbone that can maintain hundreds of kilobases of sequence in *E. coli* at low copy. While these plasmids can be used to clone large DNA fragments, they are inconvenient to work with due the large vector size and low copy number. Smaller plasmids such as pPtPuc3 (a derivative of the pUC19 plasmid) are easier to clone and can be advantageous when over-expressing proteins in *E. coli*. However, diatom genes of interest may be toxic to donor *E. coli* cells when highly expressed, and the metabolic energy required to maintain many plasmids can cause growth impairments compared to lower copy alternatives (Jones et al., 2000). Furthermore, high-copy number plasmids may become unstable leading to unwanted sequence modifications in *E. coli* (Green and Sambrook, 2012). To address some of these vector issues, the plasmid pBR322 was developed to be a medium copy number alternative and has since undergone several improvements, becoming a widely popular plasmid vector (Sutcliffe, 1979; Watson, 1988). Plasmid pBR322 shares the MB1 origin of replication with pUC19 but also contains the *Rop* gene, which regulates plasmid copy number to lower levels (Sutcliffe, 1979).

We successfully modified this plasmid to create the pPtPBR plasmids, derivatives capable of transfer to *P. tricornutum via* bacterial conjugation. We constructed the derivative plasmid pPtPBR1 (Figure 1A), which we showed can be stably maintained as an episome in *P. tricornutum* (Figure S1B in Supplementary Material). While dissecting the yeast-derived sequence required for episome maintenance in *P. tricornutum*, we found that pPtPBR derivatives containing the *CEN6, ARSH4*, and a truncated version of the *HIS3* sequence encompassing the low GC portion in a contiguous sequence resulted in a higher number of exconjugant colonies than versions containing the entire *HIS3* sequence (Figure 2). As a result, we developed episomes that are smaller, potentially easier to assemble, and more efficient in terms of exconjugant yields. We also constructed pPtPBR2 (Figure 1B), which does not have the ability to replicate as an episome and can be used as a negative control in conjugation experiments.

One major obstacle to genetic engineering of algae *via* biolistics is random genomic integration of transgenes and markers, leading to differences in gene expression and phenotypes between clones obtained from the same experiment. In addition, mapping of the integration sites is time-consuming, with possible epigenetic effects on transcription adding a layer of complexity. With the diatom episomes described herein, a mechanism is provided for the introduction of expression cassettes and markers at ploidy equivalent to native chromosome levels, while reducing the possible effects of gene disruption and deletion upon random genomic integration. Some potential applications include more meaningful and reproducible measurements of promoter and terminator strengths and greater consistency in controlling inducible promoters. Additionally, the episome allows for what could be a stable platform for the delivery of heterologous genes and pathways, as at least 50 kb of DNA can be maintained within the episome (Karas et al., 2015). Phenotypes resulting from introduction of heterologous genes into *P. tricornutum* could furthermore be calibrated more efficiently when screened across a large number of clones. Finally, the episome and the methods developed in this study could enable large-scale screening of genomic libraries in forward genetics approaches.

### Yeast Centromere and Origin of Replication Sequences Do Not Function Orthologously in *P. tricornutum*

This study begins to investigate the role that yeast artificial chromosome maintenance sequences play in diatom episome maintenance. The identity and characteristics of elements required for native diatom chromosomal replication and maintenance are unknown, but our results here suggest that these requirements differ from yeast episome maintenance. In yeast, both a centromere sequence and a sequence functioning as an origin of replication are necessary for a plasmid to be stably maintained (Murray and Szostak, 1983). In early work developing yeast artificial chromosomes, some initial cloned sequences of yeast centromeres could act alone to maintain plasmids because the sequence also contained a weak replication origin, but both functions are still required. Multiple centromeres lead to unstable dicentric plasmids, while multiple replication origins tend to enhance maintenance especially for large, cloned, and high GC sequences (Karas et al., 2013). We found that the yeast centromere and yeast replication origin sequences, when cloned individually, do not enable efficient diatom episome maintenance (Figure 2A). When the yeast centromere and yeast replication origin sequences were cloned on the same plasmid, but spatially separated, they did not allow episome maintenance in *P. tricornutum* (Figure 2A). Such a situation in yeast should lead to a stable plasmid; therefore, we conclude that these sequences do not function the same in *P. tricornutum* and yeast, and that the proximity of these yeast sequences is important for achieving the high levels of diatoms colonies after conjugation resulting from episomal maintenance.

It is possible that the *ARSH4* element alone enables episome maintenance in *P. tricornutum*, as we were able to rescue a small number of *E. coli* colonies after transformation of *P. tricornutum*-extracted DNA. One possible explanation for this is that the episomes containing *ARSH4* only are inefficiently maintained and slow-growing, and there is strong selection for chromosomal integration of the selectable marker at some point during colony formation. Thus, pPtPBR4 exconjugant *P. tricornutum* colonies may be composed of a mixture of chromosomal integrants and inefficiently maintained episomes. This could explain the smaller number of *E. coli* colonies rescued using DNA extracted from these colonies. Alternatively, it is possible that rearrangements of the plasmid (e.g., insertions of genomic DNA, recombination) may be responsible for our observation; however, this is unlikely given that pPtPBR2, 3, and 5 *P. tricornutum* exconjugant lines did not yield a single *E. coli* colony during attempts at episome rescue. Our experiments did not rule out the possibility that the *ARSH4* sequence alone could still contribute to diatom episome maintenance, whether as an origin of replication or by another mechanism. Regardless, while the mechanism of inefficient episomal maintenance by *ARSH4*-only plasmids is still unclear, episomes containing only *ARSH4* are not practical, as substantially fewer exconjugant lines are produced compared to the CAH elements combined.

Conjugative transfer of a plasmid containing two sets of adjacent *CEN6*-*ARSH4* sequences on different parts of the plasmid resulted in a similar number of diatom exconjugant colonies as the pPtPBR1 plasmid containing the entire CAH sequence. In yeast episomes, two replication origin sequences are not problematic and are in fact required for maintenance of large plasmids. However, two centromeric regions on a single centromeric plasmid creates an unstable dicentric plasmid (Mann and Davis, 1983). Here, we observe that two copies of a yeast *CEN6* sequence on different parts of the plasmid is at least as efficient in terms of diatom colony yield as only one copy of the *CEN6*. This lends further evidence to the possibility that the sequences are functioning differently in diatoms than in yeast.

### Low GC Content Appears to Drive Episome Maintenance in Diatoms

An emerging pattern while testing the efficiency of pPtPBR1 through pPtPBR9 was that sequences of low GC DNA longer than 100 bp were a common feature of episomes that could be maintained in *P. tricornutum*. For example, while *CEN6, ARSH4*, and *HIS3* were each individually inefficient in establishing episomes, combinations of *CEN6*-*ARSH4* (513-bp) and *ARSH4*-*HIS3* (588-bp) allowed for episome maintenance and maximal conjugation efficiency. One hypothesis stemming from this result is that a critical length of low GC sequence for episome maintenance is at least 500 bp since the 388-bp *ARSH4* sequence was not sufficient to establish robust episomes. The hypothesized GC content threshold required for episome maintenance is unknown, but the fact that the *CEN6, ARSH4*, and 5′ region of *HIS3* are all under ~32% GC (Figures 1A and 2A) may point to this level as critical. While GC content alone may define maintenance function in a sequence, alternatively, there may be specific sequence motifs or patterns responsible for episome maintenance that happen to occur more frequently in low GC content sequences. We could not, however, identify any such patterns in our analyses of the sequences.

*Phaeodactylum tricornutum* is capable of producing histidine, and there is no predicted functional role for the *HIS3* gene in diatoms. We observed that the 3′ region of the *HIS3* sequence contained a relatively high GC content compared to the first 200 bp adjacent to the *ARSH4* sequence, which was low in GC content (Figure 2B). To strengthen our hypothesis that low GC content plays some role in episome maintenance, we examined variations of the CAH sequence with modified *HIS3* sequences. Dissecting the *HIS3* sequence revealed that only a portion of this sequence was necessary for episome replication. The plasmids pPtPBR10-12 contained the *CEN6*-*ARSH4* regions as well as some of the low GC content sequence adjacent to the *ARSH4* sequence but lacked the relatively high GC content portion of the *HIS3* sequence. These three constructs resulted in a high number of exconjugant colonies, and, in fact, yielded more than pPtPBR1 containing the full CAH sequence. It is unclear why the removal of the 3′, high GC region of the *HIS3* sequence would increase conjugation efficiency. Possible explanations include: (1) the high-GC content region of the *HIS3* sequence is superfluous, and a smaller episome is more effectively transferred and maintained, (2) the high-GC region of *HIS3* directly decreases conjugation efficiency, regardless of plasmid size, or (3) the position of the CAH sequence in the episome in relation to adjacent sequences is altered, which may affect efficiency.

Low GC content has previously been observed to play an important role in episome and chromosome maintenance in eukaryotic organisms. Centromeres of the protist parasite *Plasmodium*, the causative agent of malaria, have been identified and used to construct artificial *Plasmodium* chromosomes (Bowman et al., 1999; Iwanaga et al., 2010, 2012). These centromere regions consist of 2.3–3.5 kb of extremely low GC content DNA (less than 2% GC), which is considerably lower than the average for the native nuclear chromosomes (Iwanaga et al., 2010, 2012). There was no clear origin of replication identified for the *Plasmodium* artificial chromosomes, and the authors hypothesized that the centromere origin functioned as both the centromere and replication origin. Although the sequences required for *P. tricornutum* episome maintenance were smaller (more than ~500 bp) than the *Plasmodium* sequences, they were similarly lower in GC than the nuclear chromosome average; diatom episome maintenance sequences were ~28–32% GC relative to 47% for *P. tricornutum* nuclear chromosomes (Bowler et al., 2008; Karas et al., 2013). It is possible that the mechanism is similar in that the same sequence can serve both as an origin of replication and as a centromere. Low GC was also found to be a defining feature of centromeres in the red alga *Cyanidioschyzon merolae* (Maruyama et al., 2008; Kanesaki et al., 2015). In this organism, each of the 20 chromosomes was found to have a distinct low GC region that functioned as a centromere and recruited the centromeric histone CENP-A (Kanesaki et al., 2015). These studies support the possibility that low GC content may play an important role in episome maintenance and, perhaps, in diatom DNA replication in general.

### Modified Conjugation Protocols Reduce Time and Materials Required, and Increase Exconjugant Yield

Our goals in testing variations of the conjugation protocol were twofold: (1) to improve exconjugant yield and (2) to eliminate the unnecessary expense of time and materials. A major benefit of using conjugative DNA transfer for diatom genetic manipulation is that the method is widely accessible and does not require expensive equipment such as a biolistic particle delivery system. We sought to make it even more accessible and effective and to provide a method to perform the conjugations in high-throughput format. We found that inclusion of 5% LB medium in the 1/2 × L1-agar plates used to grow the diatoms before conjugation improved exconjugant yield compared to growth on unsupplemented 1/2 × L1-agar plates after normalization for equal diatom cell counts. Additionally, we were able to reduce both time and material required to obtain exconjugants. A multi-well plate-based high-throughput method, where there is no resuspension of diatoms prior to conjugation and recovery, led to a reduced usage of materials and time transferring cultures while still leading to a high number of exconjugants. We identified optimal and sufficient plating protocols for the diatoms both before conjugation and after selection, as well as *E. coli* OD_600_ measurements. These data will allow users of the conjugation protocol to optimize conjugation procedures for their experiments based on the time and material resources available to them and the number of required diatom exconjugant colonies.

Culturing diatoms on 1/2 × L1-agar plates supplemented with 5% LB medium led to faster diatom growth and also a higher number of exconjugants after adjusting for cell number (Figure S2 in Supplementary Material). We hypothesize that the improved growth and conjugation efficiency observed are a result of the LB supplement to diatom growth, which may have led to changes in diatom cell physiology. Though *P. tricornutum* is generally thought to be autotrophic, there have been studies suggesting that these diatoms may display some heterotrophic tendencies (Cerón García et al., 2005; Hayward, 2009; Ukeles and Rose, 1976). The possibility of growing diatoms directly on 5% LB plates prior to conjugation eliminates the need to resuspend and replate diatoms prior to the conjugation procedure, which could possibly cause unnecessary stress to the cells and avoids an additional plating step that requires time and materials. Based on our results, it may also increase conjugation efficiency.

Exconjugant colonies were successfully obtained using diatoms plated the day before conjugation. The finding in Karas et al. (2015) that liquid diatom cultures could also be used prior to conjugation but with lower efficiency suggests that plating diatoms prior to conjugation does provide a benefit in terms of total colony number. Here, we show that the number of days prior to conjugation that diatoms are plated is not important, at least when plating on 1/2 × L1 plates containing 5% LB, meaning the entire protocol can be completed faster and with equal efficiency. Recovery time prior to plating exconjugants on selection was an important factor in exconjugant colony yield but could still be shortened compared to the previously published protocol (Karas et al., 2015). Recovery for 2 days prior to selective plating resulted in the highest number of colonies; however, the 50–100 diatom colonies resulting from a recovery period of only 1 day would be more than ample for many applications (e.g., a protein expression or promoter validation assay). When conjugating libraries of sequence variants shorter recovery time may increase library diversity, as many of the colonies on the *t* = Day 2 recovery plate could be clones of earlier conjugants and not novel and unique colonies.

## Conclusion

We tested the individual functional elements of the yeast-derived *CEN6-ARSH4-HIS3* to identify the region of the sequence that allowed it to confer episomal maintenance in diatoms. We found that low GC fragments of the *CEN6-ARSH4-HIS3* sequence that were contiguous and greater than 500-bp were required to support robust episome maintenance. We also further optimized the conjugation processes for *P. tricornutum* and developed a higher throughput small-scale protocol. Culturing diatoms on 1/2 × L1-agar supplemented with 5% LB medium led to faster diatom growth and a higher number of exconjugant colonies after adjusting for diatom cell number. Small-scale conjugations in 12-well plates could be performed to reduce materials required for conjugation when large numbers of colonies are not required. By refining conjugation methodology and elucidating additional features of episome maintenance sequences, this study contributes toward future advances in diatom molecular biology.

## Author Contributions

RD and PW designed research; RD, VB, and PW performed research; RD, VB, and PW analyzed data; and RD, VB, CD, AA, and PW wrote the paper.

## Conflict of Interest Statement

The authors declare that the research was conducted in the absence of any commercial or financial relationships that could be construed as a potential conflict of interest.
