# Supplementary material for: Refinement of the Diatom Episome Maintenance Sequence and Improvement of Conjugation-Based DNA Delivery Methods
Source: Front Bioeng Biotechnol. 2016 Aug 8;4:65. doi: 10.3389/fbioe.2016.00065 (PMC4976089; doi:10.3389/fbioe.2016.00065)
Supplement: Supplementary file 1 [file Table_1.DOCX]

**Supplementary Table 1.** Extended protocol for conjugation experiments, including potential modifications and a higher-throughput alternative.

**Conjugation Protocol**

**Summary:** The conjugation protocol is conducted in three general stages (diagrammed in Figure 3 of the manuscript): 1) preparation prior to the conjugation experiment, 2) the conjugation and recovery period, and 3) selection of ex-conjugants. The first stage consists of constructing and confirming the episomes relevant for a particular experiment, transforming the episomes into *E.coli* strains containing a conjugative plasmid, and preparing this *E.coli* strain as well as the diatom cells for the conjugation protocol. The second stage is the conjugation procedure itself, and the transfer of conjugation reactions to diatom culturing conditions for recovery. The third stage consists of replating the conjugation reaction on appropriate selective medium to obtain ex-conjugant colonies, and methods for screening for successful transfer of the complete episome.

**1. Prior to Conjugation**

A) Episome construction and *E.coli* conjugation strain engineering

- Construct and screen the episome for a particular experiment (also called the “cargo plasmid”) using desired cloning methods.
- Epsiomes can be constructed by inserting DNA of interest into one of the established episomal vectors (pPtPBR 1 or 11, pPtPUC3, or p0521s), or by inserting the diatom maintenance sequence and the origin of transfer into a different vector of choice.
- Confirmed episome should then be transformed into an *E. coli* strain containing the conjugative plasmid pTA-MOB (GentR). Note: The conjugative plasmid pRL443 also enables transfer of the cargo plasmid, but is itself transferable, resulting in the combination of cargo and conjugative plasmids and fewer correct ex-conjugant lines.

B) Plating and preparation of *Phaeodactylum tricornutum* (Pt) cells prior to conjugation

- 4 days prior to conjugation, centrifuge mid to late exponential growth stage Pt cultures (typical concentration range of 5 x 10^6^-1 x 10^7^ cells mL^-1^) at 3000*g.*
- Remove supernatant and resuspend in ~1 mL of L1 medium.
- Adjust cell concentration to 1.0x 10^8^ mL^-1^ and plate 250 µL on ½xL1 1% agar plates.
- Allow plates to dry in a laminar flow hood until no visible liquid remains (~5-10 min.), then immediately transfer to diatom culturing conditions for 4 days. Note: Typically, ~2 plates are prepared for every 3 conjugation reactions planned.
- Higher-throughput alternative (12-well plate plating protocol): Extensively dry (~ 1 hour in a laminar flow hood) 12-well plates containing ~3.5 mL of ½xL1-5%LB agar per well. Add ~50 µL of the adjusted diatom culture to each well and spread either by rotating the 12-well plate or using a sterile spreader or loop. Allow plates to dry briefly, and transfer to diatom culturing conditions for 1-4 days.
- Modifications:
  - L1 5% LB plates can also be used at this step and can increase diatom cell numbers by ~ 3 fold.
  - Diatoms can be plated less than 4 days prior to conjugation, including up to the day before conjugation, though higher cell densities should be plated in those situations such that similar numbers of diatom cells are present. A doubling time of ~1x per day is typically assumed (e.g. on the day before conjugation, plate 250 µL of 4 x10^8^ cells mL^-1^).
  - Liquid diatom cultures can also be used for conjugations, but yield fewer ex-conjugants than plated diatoms (~25% ex-conjugant yield of pre-plated diatoms).

C) Preparation of *E. coli* conjugation strain prior to conjugation

- Start an overnight culture of the *E.coli* strain containing the conjugative plasmid and the cargo plasmid the night before the conjugation experiment (shaking at 225rpm, 37°C).
- Grow in LB and include appropriate antibiotics (e.g., for a pPtPBR cargo plasmid and pTA-MOB conjugative plasmid, use Amp/Tet/Gm)
  - Higher-throughput alternative (12-well plate plating protocol): Same as regular protocol

**2. Conjugation and Recovery:**

A) Preparation of *E. coli* cells

- Use a dense overnight culture to inoculate a fresh 25 mL *E. coli* culture with a 1:50 dilution.
- Grow in a baffled flask, shaking at 225 rpm and 37°C, to an OD_600_ of 0.8-1.6 (about 3-4 hours).
- Spin down for 10 min at 3000*g*.
- Remove all supernatant and resuspend cell pellet by gently detaching the cell pellet, and very gently stirring and pipetting in 250 µL of SOC medium.
  - Higher-throughput alternative (12-well plate plating protocol): Option to grow a smaller volume of *E.coli* culture to the same OD, since less volume is required. Otherwise this step is the same as regular protocol
  - Note: After concentrating *E. coli*, it is important to remove all excess supernatant as it contains antibiotics that could potentially alter the conjugation reaction.

B) Preparation of Pt cells

- On the day of the conjugation experiment, add 500 µl of L1 medium to the diatom plate and scrape cells using a sterile spreader.
- Transfer resuspended cells to a microcentrifuge tube, and pipette gently to achieve a homogenous mixture.
- Adjust cells concentration to ~5.0 x 10^8^ mL^-1^. This step should be done very close to the time that the *E. coli* is pelleted and resuspended (see below) so that both are concentrated for a minimal amount of time prior to the conjugation.
  - Higher-throughput alternative (12-well plate plating protocol): this step is not required.
  - Notes:
    - Counting resuspended cells may be difficult to count due to clumping, however the final diatom concentration is flexible.
    - Cells plated on ½xL1-5%LB agar will come off more easily than those plated on ½xL1 alone.

C) Conjugation

- Mix 200 µL of Pt cells with 200 µl of *E.coli* cells (both concentrated as above) in a microcentrifuge tube by gently pipetting ~6-7 times until mixture is homogenous.
- Plate cells on ½xL1-5% LB agar plates. Note: since cells will be resuspended after recovery, the cell mixture is plated centrally and not all the way to the petri dish edge.
- Incubate plates for 90 minutes at 30^o^C in the dark, then move to diatom growth conditions to allow for a 1-2 day recovery period.
- Controls: With each conjugation we typically run a positive control (pPtPBR1 or 11) and a Pt only control (Pt mixed with 200 µL SOC rather than the *E. coli* strain).
  - Higher-throughput alternative (12-well plate plating protocol): Spot 50 µL of concentrated *E.coli* culture directly on top of the pre-plated diatom cells. Distribute *E.coli* across the surface area of the diatoms by rotating the plate. Allow *E. coli* culture to dry until no visible liquid remains (~10-15 minutes), rotating plate occasionally to redistribute *E. coli* cells. *E. coli* cells may also be gently mixed with the diatom cells at this stage using a sterile spreader or loop, which may decrease drying time and potentially increase colony yield due to a more homogenous culture mixture. After allowing cells to dry, incubate plates for 90 minutes at 30^o^C in the dark, then move to diatom growth conditions to allow for a 1-2 day recovery period.

**3) Selection**

A) Replating on selective medium

- After 1-2 days, add 1 mL of L1 medium to the reaction plate and resuspend cells using a sterile cell spreader.
- Tilt plate and transfer reaction to a microcentrifuge tube and adjust volume if necessary.
- Homogenize by gently pipetting, and plate 200 µl of the resuspended cells onto very dry (>1 hour drying time in a laminar flow hood) ½xL1-agar plates containing phleomycin 20 µg mL^-1^.
- Colonies will become visible in ~1 week, and should be large enough for selection by ~9-10 days.
  - Higher-throughput alternative (12-well plate plating protocol): Pipette ~500 µL of L1 medium into a well containing a reaction, and resuspend cells using a sterile spreader or loop. Plate entire reaction on a very dry (>1 hour drying open in a laminar flow hood) ½xL1-agar plates containing phleomycin 20 µg mL^-1^. For efficient reactions, 12-well plates can also be used for selecting ex-conjugants.
  - Notes:
    - For highly efficient reactions (e.g., conjugation of pPtPBR1 or 11), only a fraction of the reaction need be plated for ex-conjugant selection. For example, resuspended cells may be further diluted in L1 to a total volume of 1.5 mL, and 300 µL of the reaction plated.
    - Plating a less-dense cell resuspension allows diatom cells without antibiotic resistance to die faster, making ex-conjugant colonies easier to detect and isolate earlier.
    - Additional antibiotics, such as kanamycin (typically 50 µg mL^-1^) or chloramphenicol (typically 10-20µg mL^-1^), can also be added to the ½xL1-agar plates containing phleomycin in order to help rid diatom cultures of *E. coli* or other bacteria that may still be present.

B) Ex-conjugant Screening

- Ex-conjugant colonies are typically patched on selective medium multiple times to avoid possible carryover of excess plasmid from lysed *E. coli*.
- Prior to screening, Pt colonies are streaked on LB medium plates and incubated at 37°C in the dark overnight to confirm the absence of *E. coli*.
- Episome DNA is extracted from Pt cells using the method described in Karas et al. 2015.
- Once extracted, DNA is transformed into a common *E. coli* strain and selected on LB medium containing anti-biotics specific to the cargo plasmid.
- The transformed *E. coli* culture can then be used to obtain a clean plasmid DNA preparation followed by confirmation of the correct episome by the desired method (e.g., diagnostic restriction digest or sequencing). Note: ~ ½ of the episomes recovered are typically the correct sequence, while others may contain either DNA additions or deletions.
