# Supplementary material for: Refinement of the Diatom Episome Maintenance Sequence and Improvement of Conjugation-Based DNA Delivery Methods
Source: Front Bioeng Biotechnol. 2016 Aug 8;4:65. doi: 10.3389/fbioe.2016.00065 (PMC4976089; doi:10.3389/fbioe.2016.00065)
Supplement: Supplementary file 2 [file Table_2.DOCX]

**Supplementary Table 2:** Strains and plasmids used in this study.

| **Strain or Plasmid** | **Genotype or features** | **Use in this study** | **Reference or source** |
| --- | --- | --- | --- |
| **Strains** | | | |
| *P. tricornutum* CCMP 632 | Wild type diatom *P. tricornutum* | Recipient of gene transfer from *E. coli* | NCMA Culture Collection, Bowler et al. 2008 |
| *E.coli* Epi300 | *F^-^ mcrA Δ(mrr-hsdRMS-mcrBC) Φ80dlacZΔM15 ΔlacX74 recA1 endA1 araD139 Δ(ara, leu)7697 galU galK λ^-^ rpsL (Str^R^) nupG trfA dhfr* | Plasmid cloning, donor strain for conjugative transfer to *P. tricornutum,* episome recovery | Epicentre, Madison WI, USA |
| *E. coli* NEB5 alpha | *fhuA2 Δ(argF-lacZ)U169 phoA glnV44 Φ80 Δ(lacZ)M15 gyrA96 recA1 relA1 endA1 thi-1 hsdR17* | Plasmid cloning, donor strain for conjugative transfer to *P. tricornutum,* episome recovery | New England Biolabs, Ipswich, MA |
| **Plasmids** | | | |
| pBR322 | AmR, TetR, *oriT*, *RopA* | Source plasmid for backbone of cargo plasmids | Sutcliffe et al. 1979 |
| pRL2948a | CmR, EmR, *sacB, oriT* | Source plasmid for OriT of cargo plasmids | C.P Wolk, unpublished data, Weyman et al. 2011? |
| pTA-MOB | GmR, *Tra1, Tra2,* *parABCDE*, Ctl, *trfA, rep* (pBBR1) | Non-mobile conjugative plasmid used to transfer shuttle (cargo) vector from *E. coli* to *P. tricornutum* | Strand et al. 2014 |
| p0521s | CmR, OriT, URA3, ShBle, pCC1BAC-Lcyeast, *CEN6-ARSH4-HIS3* | Source plasmid for *CEN6-ARSH4-HIS3* sequence | Karas et al. 2015 |
| pPtPUC4-Km-IceuI | KmR, *OriT* | Source plasmid for I-CeuI restriction site in pPTPBR2 | This study |
| pPtPBR1 | AmR, TetR, ShBle, o*riT, RopA*, *CEN6-ARSH4-HIS3* | Test episome maintenance sequence | This study (NCBI Accession number, deposited on addgene) |
| pPtPBR2 | AmR, TetR, ShBle, *oriT, RopA* | Test episome maintenance sequence | This study (NCBI Accession number, deposited on addgene) |
| pPtPBR3 | AmR, TetR, ShBle, *oriT, RopA,* *CEN6* | Test episome maintenance sequence | This study |
| pPtPBR4 | AmR, TetR, ShBle, *oriT, RopA,* *ARSH4* | Test episome maintenance sequence | This study |
| pPtPBR5 | AmR, TetR, ShBle, *oriT, RopA*, *HIS3* | Test episome maintenance sequence | This study |
| pPtPBR6 | AmR, TetR, PhleoR, *oriT, RopA*, *CEN6-ARSH4* (adjacent in plasmid) | Test episome maintenance sequence | This study |
| pPtPBR7 | AmR, TetR, PhleoR, *oriT, RopA*, *CEN6-ARSH4* (not adjacent in plasmid) | Test episome maintenance sequence | This study |
| pPtPBR8 | AmR, TetR, PhleoR, *oriT, RopA,* *CEN6-ARSH4* (adjacent, with two copies in plasmid) | Test episome maintenance sequence | This study |
| pPtPBR9 | AmR, TetR, PhleoR, *oriT, RopA*, *ARSH4-HIS3* | Test episome maintenance sequence | This study |
| ptPBR10 | AmR, TetR, PhleoR, *oriT, RopA*, *CEN6-ARSH4*- first 100bp of *HIS3* | Test episome maintenance sequence | This study |
| pPtPBR11 | AmR, TetR, PhleoR, *oriT, RopA*, *CEN6-ARSH4*- first 200bp of *HIS3* | Test episome maintenance sequence | This study (NCBI Accession number, deposited on addgene) |
| pPtPBR12 | AmR, TetR, PhleoR, *oriT, RopA*, *CEN6-ARSH4*- first 300bp of *HIS3* | Test episome maintenance sequence | This study |
