# Supplementary material for: Refinement of the Diatom Episome Maintenance Sequence and Improvement of Conjugation-Based DNA Delivery Methods
Source: Front Bioeng Biotechnol. 2016 Aug 8;4:65. doi: 10.3389/fbioe.2016.00065 (PMC4976089; doi:10.3389/fbioe.2016.00065)
Supplement: Supplementary file 3 [file Table_3.DOCX]

**Supplementary Table 3:** Names and sequences of primers used in this study

| **Primer** | **Sequence** |
| --- | --- |
| amprev | CGTTAAGGGATTTTGGTCATGAG |
| CAHset1 | CTTTGGTTTCACAGTCAGGAATAACACTAGCTCGTCTTCAAAGACTCTAGGGGGATCGCC |
| CAHset10 | CACTGGCCGTCGTTTTACAA |
| CAHset2 | TTACGCCAAGCTTGCATGCCTGCAGGTCGACTCTAGAGGGATCGCTTGCCTGTAACTTAC |
| CAHset3 | ACAGTCAGGAATAACACTAGCTCGTCTTCAATCACGTGCTATAAAAATAATTATAATTTA |
| CAHset4 | ATTACGCCAAGCTTGCATGCCTGCAGGTCGACTCTAGAGGGATCGCTTGCCTGTAACTTA |
| CAHset6 | CAGCAAAGTAGTAGCTGCTCCAGTTCTCCCTAGTACACTCTATATTTTTTTATGCCTCGG |
| CAHset9 | TGAAGACGAGCTAGTGTTATTCCTG |
| CAHtest-1 | CTTCAGAAGCGTGCTATCGA |
| CAHtest1 | CTTCAGAAGCGTGCTATCGA |
| CAHtest2 | TGTGAGCGGATAACAATTTCAC |
| Cencheck1 | GAAAAAATAGTTTTTGTTTTCCGAAG |
| dCA-3 | ACAGTCAGGAATAACACTAGCTCGTCTTCATAGTACACTCTATATTTTTTTATGCCTCGG |
| endropF | ACGACCACGCTGATGAGCTTT |
| Insert-F | CCGTCAGGGCGCGTCAGCGGGTGTTGGCGGGTGTCGGGGCGGATCGTCTTGCCTTGCTCG |
| Insert-R | CGACCGAGCGCAGCGAGTCAGTGAGCGAGGAAGCGGAAGAGATGCCTGCAGGTCGACTCT |
| mid tet | CGCCGCACTTATGACTGTCT |
| pBR322-F | CTCTTCCGCTTCCTCGCTC |
| pBR322-R | CGCCCCGACACCCGCCAAC |
| PBRnanoluc 4 | TGCCTGACTGCGTTAGCAA |
| PBRnanoluc 5 | TCATGTTTGACAGCTTATCATCG |
| pPtPBR10,11,12-BB2 | AAATTATAATTATTTTTATAGCACGTGATTGAAGACGAGCTAGTGTTATTCCTGACTGT |
| pPtPBR10,11,12-Ins1 | CAGTCAGGAATAACACTAGCTCGTCTTCAATCACGTGCTATAAAAATAATTATAATTT |
| pRED8_CA/CA_BB | AAATTATAATTATTTTTATAGCACGTGATCACCCGTGGCCAGGACCCAACGCTGCCCGA |
| pRED8_CAInsert_G1 | TCGGGCAGCGTTGGGTCCTGGCCACGGGTGATCACGTGCTATAAAAATAATTATAATTT |
| prehis | GCGCGTGTAAGTTACAGGC |
| pretet | GTGCCACCTGACGTCTAAGAA |
| ptpBR10_BB1 | TTTTTTCTTAGCGATTGGCATTATCACTCTAGAGTCGACCTGCAGGCATCTCTTC |
| ptpBR10_Ins2 | GAAGAGATGCCTGCAGGTCGACTCTAGAGTGATAATGCCAATCGCTAAGAAAAAA |
| ptpBR11_BB1 | ATAAACGAAGGCAAAGATGACAGAGCAGACTCTAGAGTCGACCTGCAGGCATCTCTTCC |
| ptpBR11_Ins2 | AAATTATAATTATTTTTATAGCACGTGATTGAAGACGAGCTAGTGTTATTCCTGACTGT |
| ptpBR12_BB1 | GCGATAGAGCACTCGATCTTCCCAGAAAACTCTAGAGTCGACCTGCAGGCATCTCTTC |
| ptpBR12_Ins2 | GAAGAGATGCCTGCAGGTCGACTCTAGAGTTTTCTGGGAAGATCGAGTGCTCTATCGC |
| ptpBR9_BB_Gib1 | GGAAGAGATGCCTGCAGGTCGACTCTAGAGGTCAAGTCCAGACTCCTGTGTAAAACTAC |
| ptpBR9_Ins_Gib2 | GTAGTTTTACACAGGAGTCTGGACTTGACCTCTAGAGTCGACCTGCAGGCATCTCTTCC |
| PtPuc-14d | ACGCCAAGCTTGCATGCCTGCAGGTCGACTCTAGAGGTCAAGTCCAGACTCCTGTGTAAA |
| PtPucAddCen1 | TTTTACTTTCTATTTTTAATTTATATATTTATATTAAAAAATTTAAATTATAATTATTTTTATAGCACGTGATTGAAGACGAGCTAGTGTTATTCCTGAC |
| PtPucAddCen2 | ATATAAATATATAAATTAAAAATAGAAAGTAAAAAAAGAAATTAAAGAAAAAATAGTTTTTGTTTTCCGAAGATGTAACTCTAGAGTCGACCTGCAGGCA |
| ptREP7_Ars2insert_Gib1 | CAGCGTTGGGTCCTGGCCACGGGTGAAGACTCTAGGGGGATCGCCAACAA |
| ptREP7_Ars2insert_Gib2 | TCAACGACAGGAGCACGATCATGCGGGATCGCTTGCCTGTAACTTACACG |
| ptREP7_BB_Gib1 | CGTGTAAGTTACAGGCAAGCGATCCCGCATGATCGTGCTCCTGTCGTTGA |
| ptREP7_BB_Gib2 | TTGTTGGCGATCCCCCTAGAGTCTTCACCCGTGGCCAGGACCCAACGCTG |
| ptrepinsertscrn1 | GTGGATAACCGTATTACCGCC |
| ptrepinsertscrn2 | CAGCTTGTCTGTAAGCGGA |
| seqtest3 | CTATAATGACCCCGAAGCAGG |
