# Supplementary material for: Refinement of the Diatom Episome Maintenance Sequence and Improvement of Conjugation-Based DNA Delivery Methods
Source: Front Bioeng Biotechnol. 2016 Aug 8;4:65. doi: 10.3389/fbioe.2016.00065 (PMC4976089; doi:10.3389/fbioe.2016.00065)
Supplement: Supplementary file 4 [file Table_4.DOCX]

| **Plasmid name** | **Description** | **Insert primers** | **Insert template** | **Vector primers** | **Vector template** |
| --- | --- | --- | --- | --- | --- |
| pPtPBR-1 | CEN6-ARSH4-HIS3 | InsertF+ InsertR | pPtPuc3km | pBR322-F+ pBR322-R | pBR322 |
| pPtPBR-2 | No CEN6-ARSH4-HIS3 | InsertF+ InsertR | pPtPuc4km | pBR322-F+ pBR322-R | pBR322 |
| pPtPBR-3 | CEN6 | InsertF+ InsertR | pPtPuc-km+Cen | pBR322-F+ pBR322-R | pBR322 |
| pPtPBR-4 | ARSH4 | InsertF+ InsertR | pPtPuc-km+Ars | pBR322-F+ pBR322-R | pBR322 |
| pPtPBR-5 | HIS3 | InsertF+ InsertR | pPtPuc-km+His | pBR322-F+ pBR322-R | pBR322 |
| pPtPBR-6 | CEN6-ARSH4 | InsertF+ InsertR | pPtPuc-km+CenArs | pBR322-F+ pBR322-R | pBR322 |
| pPtPBR-7 | CEN6 separated from ARSH4 | ptREP7_Ars2insert_Gib1 + ptREP7_Ars2insert_Gib2 | pPtPBR1 | ptREP7_BB_Gib1 + ptREP_BB_Gib2 | pPtPBR3 |
| pPtPBR-8 | CEN6-ARSH4 duplicated at second site in plasmid | pRED8_CAInsert_G1 + ptREP7_Ars2Insert_Gib 2 | pPtPBR6 | pRED8_CA/CA_BB + ptREP7_BB_Gib1 | pPtPBR6 |
| pPtPBR-9 | ARSH4-HIS3 | ptpBR9_Ins_Gib1 + ptpBR9_Ins_Gib2 | pPtPBR1 | ptpBR9_BB_Gib1 + ptpBR9_BB_Gib2 | pPtPBR1 |
| pPtPBR-10 | CEN6-ARSH4-HIS3(100-bp) | ptpBR10,11,12_Ins1 +ptpBR10_Ins2 | pPtPBR1 | ptpBR10,11,12_BB2 +ptpBR10_BB1 | pPtPBR1 |
| pPtPBR-11 | CEN6-ARSH4-HIS3(200-bp) | ptpBR10,11,12_Ins1 +ptpBR11_Ins2 | pPtPBR1 | ptpBR10,11,12_BB2 +ptpBR11_BB1 | pPtPBR1 |
| pPtPBR-12 | CEN6-ARSH4-HIS3(300-bp) | ptpBR10,11,12_Ins1 +ptpBR12_Ins2 | pPtPBR1 | ptpBR10,11,12_BB2 +ptpBR12_BB1 | pPtPBR1 |

**Supplementary Table 4**: Primers and plasmids templates used to construct plasmids pPtPBR1-12 using Gibson Assembly.
