# Supplementary material for: Refinement of the Diatom Episome Maintenance Sequence and Improvement of Conjugation-Based DNA Delivery Methods
Source: Front Bioeng Biotechnol. 2016 Aug 8;4:65. doi: 10.3389/fbioe.2016.00065 (PMC4976089; doi:10.3389/fbioe.2016.00065)
Supplement: Supplementary file 5 [file Table_5.DOCX]

**Supplementary Table 5:** *E. coli* colonies recovered after extracting DNA from diatom ex-conjugants and transforming into *E. coli*, including the total number of colonies recovered, and the number of colonies normalized to the DNA concentration transformed.

| **Episome**  **Construct** | ***E. coli* colonies total** | ***E. coli* colonies per ug DNA transformed** | **ng DNA/ul transformed into *E. coli*** |
| --- | --- | --- | --- |
| pPtPBR1 | 1447 | 141 | 10.28 |
| pPtPBR2 | 0 | 0 | 8.21 |
| pPtPBR3 | 0 | 0 | 4.66 |
| pPtPBR4 | 11 | 4 | 2.54 |
| pPtPBR5 | 0 | 0 | 2.94 |
| pPtPBR6 | 268 | 117 | 2.29 |
| pPtPBR7 | 12 | 5 | 2.62 |
| pPtPBR8 | 867 | 61 | 14.17 |
| pPtPBR9 | 1711 | 194 | 8.83 |
| pPtPBR10 | 1119 | 58 | 19.38 |
| pPtPBR11 | 1505 | 117 | 12.89 |
| pPtPBR12 | 1523 | 132 | 11.57 |
