# Supplementary material for: Refinement of the Diatom Episome Maintenance Sequence and Improvement of Conjugation-Based DNA Delivery Methods
Source: Front Bioeng Biotechnol. 2016 Aug 8;4:65. doi: 10.3389/fbioe.2016.00065 (PMC4976089; doi:10.3389/fbioe.2016.00065)
Supplement: Supplementary file 6 [file Table_6.DOCX]

**Supplementary Table 6:** Optimization experiments conducted in this study, including *E. coli* OD_600_ and diatom ex-conjugant colony counts for each experiment.

| **Experiment** | **Treatment** | ***E. coli* OD600** | **Diatom colony numbers** | **Dilution factor** | **Total colony number** | **Average** | **St. Dev.** |
| --- | --- | --- | --- | --- | --- | --- | --- |
| **Plating diatoms on ½xL1 plates vs. ½xL1 + 5% LB agar plates** | L1 plates | 1.6 | 495 | 5 | 2475 | 2632 | 188 |
|  | L1 plates | 1.6 | 516 | 5 | 2580 |  |  |
|  | L1 plates | 1.6 | 568 | 5 | 2840 |  |  |
|  | L1 + 5% LB plates | 1.6 | 1258 | 5 | 6290 | 6133 | 221 |
|  | L1 + 5% LB plates | 1.6 | 1246 | 5 | 6230 |  |  |
|  | L1 + 5% LB plates | 1.6 | 1176 | 5 | 5880 |  |  |
| **Plating diatoms on ½xL1 plates vs. ½xL1 + 5% LB agar plates**  **(repeated independently)** | L1 plates | 0.8 | 1746 | 1 | 1746 | 1497 | 265 |
|  | L1 plates | 0.8 | 1526 | 1 | 1526 |  |  |
|  | L1 plates | 0.8 | 1219 | 1 | 1219 |  |  |
|  | L1 + 5% LB plates | 0.8 | 3777 | 1 | 3777 | 3618 | 301 |
|  | L1 + 5% LB plates | 0.8 | 3271 | 1 | 3271 |  |  |
|  | L1 + 5% LB plates | 0.8 | 3807 | 1 | 3807 |  |  |
| **Plating diatoms of different days prior to conjugation** | 4 days prior to conjugation | 0.8 | 433 | 1 | 433 | 399 | 30 |
|  | 4 days prior to conjugation | 0.8 | 389 | 1 | 389 |  |  |
|  | 4 days prior to conjugation | 0.8 | 375 | 1 | 375 |  |  |
|  | 3 days prior to conjugation | 0.8 | 311 | 1 | 311 | 356 | 55 |
|  | 3 days prior to conjugation | 0.8 | 340 | 1 | 340 |  |  |
|  | 3 days prior to conjugation | 0.8 | 417 | 1 | 417 |  |  |
|  | 2 days prior to conjugation | 0.8 | 393 | 1 | 393 | 401 | 28 |
|  | 2 days prior to conjugation | 0.8 | 378 | 1 | 378 |  |  |
|  | 2 days prior to conjugation | 0.8 | 432 | 1 | 432 |  |  |
|  | 1 days prior to conjugation | 0.8 | 359 | 1 | 359 | 429 | 61 |
|  | 1 days prior to conjugation | 0.8 | 458 | 1 | 458 |  |  |
|  | 1 days prior to conjugation | 0.8 | 469 | 1 | 469 |  |  |
| **Plating conjugation reaction on selective plates on different days following conjugation** | Same day of conjugation | 0.8 | 2 | 1 | 2 | 2 | 2 |
|  | Same day of conjugation | 0.8 | 0 | 1 | 0 |  |  |
|  | Same day of conjugation | 0.8 | 3 | 1 | 3 |  |  |
|  | 1 day after conjugation | 0.8 | 57 | 1 | 57 | 79 | 23 |
|  | 1 day after conjugation | 0.8 | 104 | 1 | 104 |  |  |
|  | 1 day after conjugation | 0.8 | 75 | 1 | 75 |  |  |
|  | 2 days after conjugation | 0.8 | 230 | 1 | 230 | 242 | 11 |
|  | 2 days after conjugation | 0.8 | 251 | 1 | 251 |  |  |
|  | 2 days after conjugation | 0.8 | 244 | 1 | 244 |  |  |
| **Selection on plates containing phleomychin and additional antibiotics** | phleomycin only | 0.9 | 505 | 9.37 | 4732 | 4507 | 350 |
|  | phleomycin only | 0.9 | 438 | 9.37 | 4104 |  |  |
|  | phleomycin only | 0.9 | 500 | 9.37 | 4685 |  |  |
|  | phleomycin + kanamycin | 0.9 | 544 | 9.37 | 5097 | 5272 | 378 |
|  | phleomycin + kanamycin | 0.9 | 535 | 9.37 | 5013 |  |  |
|  | phleomycin + kanamycin | 0.9 | 609 | 9.37 | 5706 |  |  |
|  | phleomycin +chloramphenicol | 0.9 | 518 | 9.37 | 4854 | 4498 | 366 |
|  | phleomycin +chloramphenicol | 0.9 | 440 | 9.37 | 4123 |  |  |
|  | phleomycin +chloramphenicol | 0.9 | 482 | 9.37 | 4516 |  |  |
