# Supplementary material for: Refinement of the Diatom Episome Maintenance Sequence and Improvement of Conjugation-Based DNA Delivery Methods
Source: Front Bioeng Biotechnol. 2016 Aug 8;4:65. doi: 10.3389/fbioe.2016.00065 (PMC4976089; doi:10.3389/fbioe.2016.00065)
Supplement: Supplementary file 7 [file Table_7.DOCX]

**Supplementary Table 7.** Profiles containing DNA sequences, protein sequences, and other relevant features of the diatom episomal vectors pPtPBR1, pPtPBR2, and pPtPBR11.

LOCUS pPtPR1_sequenced.1 7979 bp DNA circular UNA 18-OCT-2007

DEFINITION Cloning vector pBR322, complete sequence.

KEYWORDS .

SOURCE Cloning vector pBR322 (unknown)

ORGANISM Cloning vector pBR322

other sequences; artificial sequences; vectors.

REFERENCE 1 (bases 1 to 4361)

AUTHORS New England Biolabs.

TITLE Direct Submission

JOURNAL Submitted (18-OCT-2007) Research Department, New England Biolabs,

240 County Road, Ipswich, MA 01938, USA

COMMENT 1. pBR322 beta-lactamase (bla, conferring ampicillin resistance)

is transcribed in vitro and in vivo both from its natural promoter

(P3) and from promoter P1, a pSC101 promoter artificially

juxtaposed with the bla sequence during construction of the pBR322

plasmid. (Ref: Brosius et al, J Biol Chem 257, 9205-9210 (1982)).

2. Corrects sequence error in tet gene deriving from the original

pSC101 sequence, present in GenBank entry (see accession J01749)

and NEB sequences dated 6/6/02 and earlier, based on sequencing of

pBR322 and pACYC184 at NEB in 2007 (Mathieu-Sheltry, V. and Cantin,

E., unpublished). Correction is C1134T, representing a Thr350Ile

correction in the expressed protein.

FEATURES Location/Qualifiers

source 1..7979

/organism="Cloning vector pBR322"

/mol_type="other DNA"

-35_signal 10..15

/note="tet (P2) promoter (clockwise); TTGACA"

-10_signal 33..38

/note="tet (P2) promoter (clockwise); TTTAAT; transcript

start 45"

-10_signal complement(44..49)

/note="P1 promoter (counter-clockwise); TAAACT"

-35_signal complement(64..75)

/note="P1 promoter -35 sequence region; transcript starts

36, 37 (complementary strand)"

CDS 86..1276

/gene="tet"

/note="tetR (confers resistance to tetracycline)"

/codon_start=1

/product="tetracycline efflux protein, class C"

/translation="MKSNNALIVILGTVTLDAVGIGLVMPVLPGLLRDIVHSDSIASH

YGVLLALYALMQFLCAPVLGALSDRFGRRPVLLASLLGATIDYAIMATTPVLWILYAG

RIVAGITGATGAVAGAYIADITDGEDRARHFGLMSACFGVGMVAGPVAGGLLGAISLH

APFLAAAVLNGLNLLLGCFLMQESHKGERRPMPLRAFNPVSSFRWARGMTIVAALMTV

FFIMQLVGQVPAALWVIFGEDRFRWSATMIGLSLAVFGILHALAQAFVTGPATKRFGE

KQAIIAGMAADALGYVLLAFATRGWMAFPIMILLASGGIGMPALQAMLSRQVDDDHQG

QLQGSLAALTSLTSIIGPLIVTAIYAASASTWNGLAWIVGAALYLVCLPALRRGAWSR

ATST"

gene 86..1276

/gene="tet"

RBS 1905..1909

/note="rop RBS"

CDS 1915..2106

/gene="rop"

/exception="alternative start codon"

/codon_start=1

/transl_table=11

/product="ROP protein"

/translation="MTKQEKTALNMARFIRSQTLTLLEKLNELDADEQADICESLHDH

ADELYRSCLARFGDDGENL"

gene 1915..2106

/gene="rop"

primer 2169..2239

/label=Insert-F

primer complement(2191..2209)

/label=pBR322-R

OriT 2210..2985

/label=OriT-pRL2948a

Insert 2210..5968

ShBle 3145..4562

/label=PtBle

CEN 4569..4685

Ars 4686..5073

HIS 5074..5945

Site join(5837..5845,5840^5841)

/site_type="restriction site"

/note="Name: Tth111I"

/note="Pattern: gacnnngtc"

/note="Number_of_matches: 1"

/note="cut_0_on_positive_strand: 2220^2221"

/note="cut_0_on_negative_strand: 2221^2222"

/note="inhibited_by: 5': N6-methyladenosine"

/note="star_activity: yes"

/note="site_type: other"

/note="restriction site"

/label=Tth111I

primer complement(5950..6009)

/label=Insert-R

primer 5969..5987

/label=pBR322-F

misc_RNA complement(6152..6704)

/note="RNAII transcript (complementary strand)"

rep_origin complement(6152..6740)

/note="pMB1 origin of replication (counter-clockwise)

(RNAII -35 to RNA/DNA switch point)"

-35_signal 6558..6563

/note="RNAI promoter (clockwise); TTGAAG"

-10_signal 6580..6585

/note="RNAI promoter (clockwise); GCTACA"

misc_RNA 6594..6701

/note="RNAI transcript"

-10_signal complement(6714..6719)

/note="RNAII promoter (counter-clockwise); CGTAAT"

-35_signal complement(6735..6740)

/note="RNAII promoter (counter-clockwise); TTGAGA"

primer 6780..6802

/label=Amp-Primer-R

CDS complement(6911..7771)

/gene="bla"

/note="ampR (confers resistance to ampicillin)"

/codon_start=1

/product="beta-lactamase"

/translation="MSIQHFRVALIPFFAAFCLPVFAHPETLVKVKDAEDQLGARVGY

IELDLNSGKILESFRPEERFPMMSTFKVLLCGAVLSRVDAGQEQLGRRIHYSQNDLVE

YSPVTEKHLTDGMTVRELCSAAITMSDNTAANLLLTTIGGPKELTAFLHNMGDHVTRL

DRWEPELNEAIPNDERDTTMPAAMATTLRKLLTGELLTLASRQQLIDWMEADKVAGPL

LRSALPAGWFIADKSGAGERGSRGIIAALGPDGKPSRIVVIYTTGSQATMDERNRQIA

EIGASLIKHW"

gene complement(6911..7771)

/gene="bla"

sig_peptide complement(7703..7771)

/gene="bla"

/note="required for secretion to the periplasm; cleaved

off to form the mature beta-lactamase protein."

RBS complement(7779..7783)

/note="bla RBS"

-10_signal complement(7815..7820)

/note="bla (P3) promoter (counter-clockwise); GAGACA;

transcript start 4188 (complementary strand)"

-35_signal complement(7836..7841)

/note="bla (P3) promoter (counter-clockwise); TTCAAA"

primer complement(7958..7977)

/label=Amp-Primer-F

ORIGIN

1 TTCTCATGTT TGACAGCTTA TCATCGATAA GCTTTAATGC GGTAGTTTAT CACAGTTAAA

61 TTGCTAACGC AGTCAGGCAC CGTGTATGAA ATCTAACAAT GCGCTCATCG TCATCCTCGG

121 CACCGTCACC CTGGATGCTG TAGGCATAGG CTTGGTTATG CCGGTACTGC CGGGCCTCTT

181 GCGGGATATC GTCCATTCCG ACAGCATCGC CAGTCACTAT GGCGTGCTGC TAGCGCTATA

241 TGCGTTGATG CAATTTCTAT GCGCACCCGT TCTCGGAGCA CTGTCCGACC GCTTTGGCCG

301 CCGCCCAGTC CTGCTCGCTT CGCTACTTGG AGCCACTATC GACTACGCGA TCATGGCGAC

361 CACACCCGTC CTGTGGATCC TCTACGCCGG ACGCATCGTG GCCGGCATCA CCGGCGCCAC

421 AGGTGCGGTT GCTGGCGCCT ATATCGCCGA CATCACCGAT GGGGAAGATC GGGCTCGCCA

481 CTTCGGGCTC ATGAGCGCTT GTTTCGGCGT GGGTATGGTG GCAGGCCCCG TGGCCGGGGG

541 ACTGTTGGGC GCCATCTCCT TGCATGCACC ATTCCTTGCG GCGGCGGTGC TCAACGGCCT

601 CAACCTACTA CTGGGCTGCT TCCTAATGCA GGAGTCGCAT AAGGGAGAGC GTCGACCGAT

661 GCCCTTGAGA GCCTTCAACC CAGTCAGCTC CTTCCGGTGG GCGCGGGGCA TGACTATCGT

721 CGCCGCACTT ATGACTGTCT TCTTTATCAT GCAACTCGTA GGACAGGTGC CGGCAGCGCT

781 CTGGGTCATT TTCGGCGAGG ACCGCTTTCG CTGGAGCGCG ACGATGATCG GCCTGTCGCT

841 TGCGGTATTC GGAATCTTGC ACGCCCTCGC TCAAGCCTTC GTCACTGGTC CCGCCACCAA

901 ACGTTTCGGC GAGAAGCAGG CCATTATCGC CGGCATGGCG GCCGACGCGC TGGGCTACGT

961 CTTGCTGGCG TTCGCGACGC GAGGCTGGAT GGCCTTCCCC ATTATGATTC TTCTCGCTTC

1021 CGGCGGCATC GGGATGCCCG CGTTGCAGGC CATGCTGTCC AGGCAGGTAG ATGACGACCA

1081 TCAGGGACAG CTTCAAGGAT CGCTCGCGGC TCTTACCAGC CTAACTTCGA TCATTGGACC

1141 GCTGATCGTC ACGGCGATTT ATGCCGCCTC GGCGAGCACA TGGAACGGGT TGGCATGGAT

1201 TGTAGGCGCC GCCCTATACC TTGTCTGCCT CCCCGCGTTG CGTCGCGGTG CATGGAGCCG

1261 GGCCACCTCG ACCTGAATGG AAGCCGGCGG CACCTCGCTA ACGGATTCAC CACTCCAAGA

1321 ATTGGAGCCA ATCAATTCTT GCGGAGAACT GTGAATGCGC AAACCAACCC TTGGCAGAAC

1381 ATATCCATCG CGTCCGCCAT CTCCAGCAGC CGCACGCGGC GCATCTCGGG CAGCGTTGGG

1441 TCCTGGCCAC GGGTGCGCAT GATCGTGCTC CTGTCGTTGA GGACCCGGCT AGGCTGGCGG

1501 GGTTGCCTTA CTGGTTAGCA GAATGAATCA CCGATACGCG AGCGAACGTG AAGCGACTGC

1561 TGCTGCAAAA CGTCTGCGAC CTGAGCAACA ACATGAATGG TCTTCGGTTT CCGTGTTTCG

1621 TAAAGTCTGG AAACGCGGAA GTCAGCGCCC TGCACCATTA TGTTCCGGAT CTGCATCGCA

1681 GGATGCTGCT GGCTACCCTG TGGAACACCT ACATCTGTAT TAACGAAGCG CTGGCATTGA

1741 CCCTGAGTGA TTTTTCTCTG GTCCCGCCGC ATCCATACCG CCAGTTGTTT ACCCTCACAA

1801 CGTTCCAGTA ACCGGGCATG TTCATCATCA GTAACCCGTA TCGTGAGCAT CCTCTCTCGT

1861 TTCATCGGTA TCATTACCCC CATGAACAGA AATCCCCCTT ACACGGAGGC ATCAGTGACC

1921 AAACAGGAAA AAACCGCCCT TAACATGGCC CGCTTTATCA GAAGCCAGAC ATTAACGCTT

1981 CTGGAGAAAC TCAACGAGCT GGACGCGGAT GAACAGGCAG ACATCTGTGA ATCGCTTCAC

2041 GACCACGCTG ATGAGCTTTA CCGCAGCTGC CTCGCGCGTT TCGGTGATGA CGGTGAAAAC

2101 CTCTGACACA TGCAGCTCCC GGAGACGGTC ACAGCTTGTC TGTAAGCGGA TGCCGGGAGC

2161 AGACAAGCCC GTCAGGGCGC GTCAGCGGGT GTTGGCGGGT GTCGGGGCGG ATCGTCTTGC

2221 CTTGCTCGTC GGTGATGTAC TTCACCAGCT CCGCGAAGTC GCTCTTCTTG ATGGAGCGCA

2281 TGGGGACGTG CTTGGCAATC ACGCGCACCC CCCGGCCGTT TTAGCGGCTA AAAAAGTCAT

2341 GGCTCTGCCC TCGGGCGGAC CACGCCCATC ATGACCTTGC CAAGCTCGTC CTGCTTCTCT

2401 TCGATCTTCG CCAGCAGGGC GAGGATCGTG GCATCACCGA ACCGCGCCGT GCGCGGGTCG

2461 TCGGTGAGCC AGAGTTTCAG CAGGCCGCCC AGGCGGCCCA GGTCGCCATT GATGCGGGCC

2521 AGCTCGCGGA CGTGCTCATA GTCCACGACG CCCGTGATTT TGTAGCCCTG GCCGACGGCC

2581 AGCAGGTAGG CCGACAGGCT CATGCCGGCC GCCGCCGCCT TTTCCTCAAT CGCTCTTCGT

2641 TCGTCTGGAA GGCAGTACAC CTTGATAGGT GGGCTGCCCT TCCTGGTTGG CTTGGTTTCA

2701 TCAGCCATCC GCTTGCCCTC ATCTGTTACG CCGGCGGTAG CCGGCCAGCC TCGCAGAGCA

2761 GGATTCCCGT TGAGCACCGC CAGGTGCGAA TAAGGGACAG TGAAGAAGGA ACACCCGCTC

2821 GCGGGTGGGC CTACTTCACC TATCCTGCCC GGCTGACGCC GTTGGATACA CCAAGGAAAG

2881 TCTACACGAA CCCTTTGGCA AAATCCTGTA TATCGTGCGA AAAAGGATGG ATATACCGAA

2941 AAAATCGCTA TAATGACCCC GAAGCAGGGT TATGCAGCGG AAGATGCCAT TCGCCATTCA

3001 GGCTGCGCAA CTGTTGGGAA GGGCGATCGG TGCGGGCCTC TTCGCTATTA CGCCAGCTGG

3061 CGAAAGGGGG ATGTGCTGCA AGGCGATTAA GTTGGGTAAC GCCAGGGTTT TCCCAGTCAC

3121 GACGTTGTAA AACGACGGCC AGTGACCATG ATTACGCCAA GCTCGAAATT AACCCTCACT

3181 AAAGGGAACA AAAGCTGGTA CCTAACAGGA TTAGTGCAAT TCGAGTTGAA TCACTGGGAA

3241 AAACATTGTC TTCTTTTTTA TATTATCATT TGCATTAGTG CTGCAGTCGT AGATACTTGT

3301 TGGTTGAAAG ACATCAGCTG GGAGGGACTG GACTAGCGTT TGGTAAGGAG ACATACCTGT

3361 TAACGTTGGT TGCAAAATTC CATTTCGCGA TTTATGTTAT CTGTAAATCC TGATTTGTCT

3421 GGAATTCTTG ATACTTCCGT TTTTTTAGAG GCCAATGATT AGCATCGGCG ATTCTCAAAA

3481 TAGCATTTTC GACATGCGGT GCTGATTTCA TAAACATAGA CAACGCTTTT ACATGTAAAA

3541 GTAACTTGCG GACTTGGAAC AGTGCTCTGT TTTTGGTGTG AACGTAACTC AGCAATATTT

3601 CTGTGCTAGC AAGGTTTTTT ATGATCGACC GAAGATCTCA AAACTCCGGG TCTTTCAACT

3661 GTCTGACTAG ACCATGTTCG TAACGTCGGA CAGCAGCTTT CGTTGTACTG GTAGAATTTC

3721 TACGTGCGAA GCACGTGTAG GCAGGTTGAA CGACGATCCC TGCCGATGGA TGGATTGGCA

3781 CGCGGCGGAA CGCTTTCGTG ATCTACACCA CCTGGATCTT CACATATCTT CGAAATCGAA

3841 AAATTAACCA AGTCGACGGT ATCGATAATA TTCTAGCTGA GGGTACCCAT GGCCAAGTTG

3901 ACCAGTGCCG TTCCGGTGCT CACCGCGCGC GACGTCGCCG GAGCGGTCGA GTTCTGGACC

3961 GACCGGCTCG GGTTCTCCCG GGACTTCGTG GAGGACGACT TCGCCGGTGT GGTCCGGGAC

4021 GACGTGACCC TGTTCATCAG CGCGGTCCAG GACCAGGTGG TGCCGGACAA CACCCTGGCC

4081 TGGGTGTGGG TGCGCGGCCT GGACGAGCTG TACGCCGAGT GGTCGGAGGT CGTGTCCACG

4141 AACTTCCGGG ACGCCTCCGG GCCGGCCATG ACCGAGATCG GCGAGCAGCC GTGGGGGCGG

4201 GAGTTCGCCC TGCGCGACCC GGCCGGCAAC TGCGTGCACT TCGTGGCCGA GGAGCAGGAC

4261 TGACCGACGC CGACCAACAC CGCCGGTCCG ACGCGGCCCG ACGGGTCCGA GGCCTCGGAG

4321 ATCTGGGCCC ATGCGGCCGC AACAACTACC TCGACTTTGG CTGGGACACT TTCAGTGAGG

4381 ACAAGAAGCT TCAGAAGCGT GCTATCGAAC TCAACCAGGG ACGTGCGGCA CAAATGGGCA

4441 TCCTTGCTCT CATGGTGCAC GAACAGTTGG GAGTCTCTAT CCTTCCTTAA AAATTTAATT

4501 TTCATTAGTT GCAGTCACTC CGCTTTGGTT TCACAGTCAG GAATAACACT AGCTCGTCTT

4561 CAGCGAGCAT CACGTGCTAT AAAAATAATT ATAATTTAAA TTTTTTAATA TAAATATATA

4621 AATTAAAAAT AGAAAGTAAA AAAAGAAATT AAAGAAAAAA TAGTTTTTGT TTTCCGAAGA

4681 TGTAAAAGAC TCTAGGGGGA TCGCCAACAA ATACTACCTT TTACCTTGCT CTTCCTGCTC

4741 TCAGGTATTA ATGCCGAATT GTTTCATCTT GTCTGTGTAG AAGACCACAC ACGAAAATCC

4801 TGTGATTTTA CATTTTACTT ATCGTTAATC GAATGTATAT CTATTTAATC TGCTTTTCTT

4861 GTCTAATAAA TATATATGTA AAGTACGCTT TTTGTTGAAA TTTTTTAAAC CTTTGTTTAT

4921 TTTTTTTTCT TCATTCCGTA ACTCTTCTAC CTTCTTTATT TACTTTCTAA AATCCAAATA

4981 CAAAACATAA AAATAAATAA ACACAGAGTA AATTCCCAAA TTATTCCATC ATTAAAAGAT

5041 ACGAGGCGCG TGTAAGTTAC AGGCAAGCGA TCCTAGTACA CTCTATATTT TTTTATGCCT

5101 CGGTAATGAT TTTCATTTTT TTTTTCCACC TAGCGGATGA CTCTTTTTTT TTCTTAGCGA

5161 TTGGCATTAT CACATAATGA ATTATACATT ATATAAAGTA ATGTGATTTC TTCGAAGAAT

5221 ATACTAAAAA ATGAGCAGGC AAGATAAACG AAGGCAAAGA TGACAGAGCA GAAAGCCCTA

5281 GTAAAGCGTA TTACAAATGA AACCAAGATT CAGATTGCGA TCTCTTTAAA GGGTGGTCCC

5341 CTAGCGATAG AGCACTCGAT CTTCCCAGAA AAAGAGGCAG AAGCAGTAGC AGAACAGGCC

5401 ACACAATCGC AAGTGATTAA CGTCCACACA GGTATAGGGT TTCTGGACCA TATGATACAT

5461 GCTCTGGCCA AGCATTCCGG CTGGTCGCTA ATCGTTGAGT GCATTGGTGA CTTACACATA

5521 GACGACCATC ACACCACTGA AGACTGCGGG ATTGCTCTCG GTCAAGCTTT TAAAGAGGCC

5581 CTAGGGGCCG TGCGTGGAGT AAAAAGGTTT GGATCAGGAT TTGCGCCTTT GGATGAGGCA

5641 CTTTCCAGAG CGGTGGTAGA TCTTTCGAAC AGGCCGTACG CAGTTGTCGA ACTTGGTTTG

5701 CAAAGGGAGA AAGTAGGAGA TCTCTCTTGC GAGATGATCC CGCATTTTCT TGAAAGCTTT

5761 GCAGAGGCTA GCAGAATTAC CCTCCACGTT GATTGTCTGC GAGGCAAGAA TGATCATCAC

5821 CGTAGTGAGA GTGCGTTCAA GGCTCTTGCG GTTGCCATAA GAGAAGCCAC CTCGCCCAAT

5881 GGTACCAACG ATGTTCCCTC CACCAAAGGT GTTCTTATGT AGTTTTACAC AGGAGTCTGG

5941 ACTTGACCTC TAGAGTCGAC CTGCGCATCT CTTCCGCTTC CTCGCTCACT GACTCGCTGC

6001 GCTCGGTCGT TCGGCTGCGG CGAGCGGTAT CAGCTCACTC AAAGGCGGTA ATACGGTTAT

6061 CCACAGAATC AGGGGATAAC GCAGGAAAGA ACATGTGAGC AAAAGGCCAG CAAAAGGCCA

6121 GGAACCGTAA AAAGGCCGCG TTGCTGGCGT TTTTCCATAG GCTCCGCCCC CCTGACGAGC

6181 ATCACAAAAA TCGACGCTCA AGTCAGAGGT GGCGAAACCC GACAGGACTA TAAAGATACC

6241 AGGCGTTTCC CCCTGGAAGC TCCCTCGTGC GCTCTCCTGT TCCGACCCTG CCGCTTACCG

6301 GATACCTGTC CGCCTTTCTC CCTTCGGGAA GCGTGGCGCT TTCTCATAGC TCACGCTGTA

6361 GGTATCTCAG TTCGGTGTAG GTCGTTCGCT CCAAGCTGGG CTGTGTGCAC GAACCCCCCG

6421 TTCAGCCCGA CCGCTGCGCC TTATCCGGTA ACTATCGTCT TGAGTCCAAC CCGGTAAGAC

6481 ACGACTTATC GCCACTGGCA GCAGCCACTG GTAACAGGAT TAGCAGAGCG AGGTATGTAG

6541 GCGGTGCTAC AGAGTTCTTG AAGTGGTGGC CTAACTACGG CTACACTAGA AGGACAGTAT

6601 TTGGTATCTG CGCTCTGCTG AAGCCAGTTA CCTTCGGAAA AAGAGTTGGT AGCTCTTGAT

6661 CCGGCAAACA AACCACCGCT GGTAGCGGTG GTTTTTTTGT TTGCAAGCAG CAGATTACGC

6721 GCAGAAAAAA AGGATCTCAA GAAGATCCTT TGATCTTTTC TACGGGGTCT GACGCTCAGT

6781 GGAACGAAAA CTCACGTTAA GGGATTTTGG TCATGAGATT ATCAAAAAGG ATCTTCACCT

6841 AGATCCTTTT AAATTAAAAA TGAAGTTTTA AATCAATCTA AAGTATATAT GAGTAAACTT

6901 GGTCTGACAG TTACCAATGC TTAATCAGTG AGGCACCTAT CTCAGCGATC TGTCTATTTC

6961 GTTCATCCAT AGTTGCCTGA CTCCCCGTCG TGTAGATAAC TACGATACGG GAGGGCTTAC

7021 CATCTGGCCC CAGTGCTGCA ATGATACCGC GAGACCCACG CTCACCGGCT CCAGATTTAT

7081 CAGCAATAAA CCAGCCAGCC GGAAGGGCCG AGCGCAGAAG TGGTCCTGCA ACTTTATCCG

7141 CCTCCATCCA GTCTATTAAT TGTTGCCGGG AAGCTAGAGT AAGTAGTTCG CCAGTTAATA

7201 GTTTGCGCAA CGTTGTTGCC ATTGCTGCAG GCATCGTGGT GTCACGCTCG TCGTTTGGTA

7261 TGGCTTCATT CAGCTCCGGT TCCCAACGAT CAAGGCGAGT TACATGATCC CCCATGTTGT

7321 GCAAAAAAGC GGTTAGCTCC TTCGGTCCTC CGATCGTTGT CAGAAGTAAG TTGGCCGCAG

7381 TGTTATCACT CATGGTTATG GCAGCACTGC ATAATTCTCT TACTGTCATG CCATCCGTAA

7441 GATGCTTTTC TGTGACTGGT GAGTACTCAA CCAAGTCATT CTGAGAATAG TGTATGCGGC

7501 GACCGAGTTG CTCTTGCCCG GCGTCAACAC GGGATAATAC CGCGCCACAT AGCAGAACTT

7561 TAAAAGTGCT CATCATTGGA AAACGTTCTT CGGGGCGAAA ACTCTCAAGG ATCTTACCGC

7621 TGTTGAGATC CAGTTCGATG TAACCCACTC GTGCACCCAA CTGATCTTCA GCATCTTTTA

7681 CTTTCACCAG CGTTTCTGGG TGAGCAAAAA CAGGAAGGCA AAATGCCGCA AAAAAGGGAA

7741 TAAGGGCGAC ACGGAAATGT TGAATACTCA TACTCTTCCT TTTTCAATAT TATTGAAGCA

7801 TTTATCAGGG TTATTGTCTC ATGAGCGGAT ACATATTTGA ATGTATTTAG AAAAATAAAC

7861 AAATAGGGGT TCCGCGCACA TTTCCCCGAA AAGTGCCACC TGACGTCTAA GAAACCATTA

7921 TTATCATGAC ATTAACCTAT AAAAATAGGC GTATCACGAG GCCCTTTCGT CTTCAAGAA

//

LOCUS pPtPBR2_sequenced 6596 bp DNA circular UNA 18-OCT-2007

DEFINITION Cloning vector pBR322, complete sequence.

KEYWORDS .

SOURCE Cloning vector pBR322 (unknown)

ORGANISM Cloning vector pBR322

other sequences; artificial sequences; vectors.

REFERENCE 1 (bases 1 to 4361)

AUTHORS New England Biolabs.

TITLE Direct Submission

JOURNAL Submitted (18-OCT-2007) Research Department, New England Biolabs,

240 County Road, Ipswich, MA 01938, USA

COMMENT 1. pBR322 beta-lactamase (bla, conferring ampicillin resistance)

is transcribed in vitro and in vivo both from its natural promoter

(P3) and from promoter P1, a pSC101 promoter artificially

juxtaposed with the bla sequence during construction of the pBR322

plasmid. (Ref: Brosius et al, J Biol Chem 257, 9205-9210 (1982)).

2. Corrects sequence error in tet gene deriving from the original

pSC101 sequence, present in GenBank entry (see accession J01749)

and NEB sequences dated 6/6/02 and earlier, based on sequencing of

pBR322 and pACYC184 at NEB in 2007 (Mathieu-Sheltry, V. and Cantin,

E., unpublished). Correction is C1134T, representing a Thr350Ile

correction in the expressed protein.

FEATURES Location/Qualifiers

source 1..6596

/organism="Cloning vector pBR322"

/mol_type="other DNA"

-35_signal 10..15

/note="tet (P2) promoter (clockwise); TTGACA"

-10_signal 33..38

/note="tet (P2) promoter (clockwise); TTTAAT; transcript

start 45"

-10_signal complement(44..49)

/note="P1 promoter (counter-clockwise); TAAACT"

Region 61..766

/label="CHECK VS NEW SEQ (PRETET)"

-35_signal complement(64..75)

/note="P1 promoter -35 sequence region; transcript starts

36, 37 (complementary strand)"

CDS 86..1276

/gene="tet"

/note="tetR (confers resistance to tetracycline)"

/codon_start=1

/product="tetracycline efflux protein, class C"

/translation="MKSNNALIVILGTVTLDAVGIGLVMPVLPGLLRDIVHSDSIASH

YGVLLALYALMQFLCAPVLGALSDRFGRRPVLLASLLGATIDYAIMATTPVLWILYAG

RIVAGITGATGAVAGAYIADITDGEDRARHFGLMSACFGVGMVAGPVAGGLLGAISLH

APFLAAAVLNGLNLLLGCFLMQESHKGERRPMPLRAFNPVSSFRWARGMTIVAALMTV

FFIMQLVGQVPAALWVIFGEDRFRWSATMIGLSLAVFGILHALAQAFVTGPATKRFGE

KQAIIAGMAADALGYVLLAFATRGWMAFPIMILLASGGIGMPALQAMLSRQVDDDHQG

QLQGSLAALTSLTSIIGPLIVTAIYAASASTWNGLAWIVGAALYLVCLPALRRGAWSR

ATST"

gene 86..1276

/gene="tet"

RBS 1905..1909

/note="rop RBS"

CDS 1915..2106

/gene="rop"

/exception="alternative start codon"

/codon_start=1

/transl_table=11

/product="ROP protein"

/translation="MTKQEKTALNMARFIRSQTLTLLEKLNELDADEQADICESLHDH

ADELYRSCLARFGDDGENL"

gene 1915..2106

/gene="rop"

primer 2169..2229

/label=Insert-F

primer complement(2191..2209)

/label=pBR322-R

OriT 2210..2985

/label=OriT-pRL2948a

Insert 2210..4585

Region 2210..2309

/label="check with new sequence against 322 and ptpbr1"

ShBle 3145..4562

/label=PtBle

primer complement(4567..4626)

/label=Insert-R

primer 4586..4604

/label=pBR322-F

misc_RNA complement(4769..5321)

/note="RNAII transcript (complementary strand)"

rep_origin complement(4769..5357)

/note="pMB1 origin of replication (counter-clockwise)

(RNAII -35 to RNA/DNA switch point)"

-35_signal 5175..5180

/note="RNAI promoter (clockwise); TTGAAG"

-10_signal 5197..5202

/note="RNAI promoter (clockwise); GCTACA"

misc_RNA 5211..5318

/note="RNAI transcript"

-10_signal complement(5331..5336)

/note="RNAII promoter (counter-clockwise); CGTAAT"

-35_signal complement(5352..5357)

/note="RNAII promoter (counter-clockwise); TTGAGA"

primer 5397..5419

/label=Amp-Primer-R

CDS complement(5528..6388)

/gene="bla"

/note="ampR (confers resistance to ampicillin)"

/codon_start=1

/product="beta-lactamase"

/translation="MSIQHFRVALIPFFAAFCLPVFAHPETLVKVKDAEDQLGARVGY

IELDLNSGKILESFRPEERFPMMSTFKVLLCGAVLSRVDAGQEQLGRRIHYSQNDLVE

YSPVTEKHLTDGMTVRELCSAAITMSDNTAANLLLTTIGGPKELTAFLHNMGDHVTRL

DRWEPELNEAIPNDERDTTMPAAMATTLRKLLTGELLTLASRQQLIDWMEADKVAGPL

LRSALPAGWFIADKSGAGERGSRGIIAALGPDGKPSRIVVIYTTGSQATMDERNRQIA

EIGASLIKHW"

gene complement(5528..6388)

/gene="bla"

sig_peptide complement(6320..6388)

/gene="bla"

/note="required for secretion to the periplasm; cleaved

off to form the mature beta-lactamase protein."

RBS complement(6396..6400)

/note="bla RBS"

-10_signal complement(6432..6437)

/note="bla (P3) promoter (counter-clockwise); GAGACA;

transcript start 4188 (complementary strand)"

-35_signal complement(6453..6458)

/note="bla (P3) promoter (counter-clockwise); TTCAAA"

primer complement(6575..6594)

/label=Amp-Primer-F

ORIGIN

1 TTCTCATGTT TGACAGCTTA TCATCGATAA GCTTTAATGC GGTAGTTTAT CACAGTTAAA

61 TTGCTAACGC AGTCAGGCAC CGTGTATGAA ATCTAACAAT GCGCTCATCG TCATCCTCGG

121 CACCGTCACC CTGGATGCTG TAGGCATAGG CTTGGTTATG CCGGTACTGC CGGGCCTCTT

181 GCGGGATATC GTCCATTCCG ACAGCATCGC CAGTCACTAT GGCGTGCTGC TAGCGCTATA

241 TGCGTTGATG CAATTTCTAT GCGCACCCGT TCTCGGAGCA CTGTCCGACC GCTTTGGCCG

301 CCGCCCAGTC CTGCTCGCTT CGCTACTTGG AGCCACTATC GACTACGCGA TCATGGCGAC

361 CACACCCGTC CTGTGGATCC TCTACGCCGG ACGCATCGTG GCCGGCATCA CCGGCGCCAC

421 AGGTGCGGTT GCTGGCGCCT ATATCGCCGA CATCACCGAT GGGGAAGATC GGGCTCGCCA

481 CTTCGGGCTC ATGAGCGCTT GTTTCGGCGT GGGTATGGTG GCAGGCCCCG TGGCCGGGGG

541 ACTGTTGGGC GCCATCTCCT TGCATGCACC ATTCCTTGCG GCGGCGGTGC TCAACGGCCT

601 CAACCTACTA CTGGGCTGCT TCCTAATGCA GGAGTCGCAT AAGGGAGAGC GTCGACCGAT

661 GCCCTTGAGA GCCTTCAACC CAGTCAGCTC CTTCCGGTGG GCGCGGGGCA TGACTATCGT

721 CGCCGCACTT ATGACTGTCT TCTTTATCAT GCAACTCGTA GGACAGGTGC CGGCAGCGCT

781 CTGGGTCATT TTCGGCGAGG ACCGCTTTCG CTGGAGCGCG ACGATGATCG GCCTGTCGCT

841 TGCGGTATTC GGAATCTTGC ACGCCCTCGC TCAAGCCTTC GTCACTGGTC CCGCCACCAA

901 ACGTTTCGGC GAGAAGCAGG CCATTATCGC CGGCATGGCG GCCGACGCGC TGGGCTACGT

961 CTTGCTGGCG TTCGCGACGC GAGGCTGGAT GGCCTTCCCC ATTATGATTC TTCTCGCTTC

1021 CGGCGGCATC GGGATGCCCG CGTTGCAGGC CATGCTGTCC AGGCAGGTAG ATGACGACCA

1081 TCAGGGACAG CTTCAAGGAT CGCTCGCGGC TCTTACCAGC CTAACTTCGA TCATTGGACC

1141 GCTGATCGTC ACGGCGATTT ATGCCGCCTC GGCGAGCACA TGGAACGGGT TGGCATGGAT

1201 TGTAGGCGCC GCCCTATACC TTGTCTGCCT CCCCGCGTTG CGTCGCGGTG CATGGAGCCG

1261 GGCCACCTCG ACCTGAATGG AAGCCGGCGG CACCTCGCTA ACGGATTCAC CACTCCAAGA

1321 ATTGGAGCCA ATCAATTCTT GCGGAGAACT GTGAATGCGC AAACCAACCC TTGGCAGAAC

1381 ATATCCATCG CGTCCGCCAT CTCCAGCAGC CGCACGCGGC GCATCTCGGG CAGCGTTGGG

1441 TCCTGGCCAC GGGTGCGCAT GATCGTGCTC CTGTCGTTGA GGACCCGGCT AGGCTGGCGG

1501 GGTTGCCTTA CTGGTTAGCA GAATGAATCA CCGATACGCG AGCGAACGTG AAGCGACTGC

1561 TGCTGCAAAA CGTCTGCGAC CTGAGCAACA ACATGAATGG TCTTCGGTTT CCGTGTTTCG

1621 TAAAGTCTGG AAACGCGGAA GTCAGCGCCC TGCACCATTA TGTTCCGGAT CTGCATCGCA

1681 GGATGCTGCT GGCTACCCTG TGGAACACCT ACATCTGTAT TAACGAAGCG CTGGCATTGA

1741 CCCTGAGTGA TTTTTCTCTG GTCCCGCCGC ATCCATACCG CCAGTTGTTT ACCCTCACAA

1801 CGTTCCAGTA ACCGGGCATG TTCATCATCA GTAACCCGTA TCGTGAGCAT CCTCTCTCGT

1861 TTCATCGGTA TCATTACCCC CATGAACAGA AATCCCCCTT ACACGGAGGC ATCAGTGACC

1921 AAACAGGAAA AAACCGCCCT TAACATGGCC CGCTTTATCA GAAGCCAGAC ATTAACGCTT

1981 CTGGAGAAAC TCAACGAGCT GGACGCGGAT GAACAGGCAG ACATCTGTGA ATCGCTTCAC

2041 GACCACGCTG ATGAGCTTTA CCGCAGCTGC CTCGCGCGTT TCGGTGATGA CGGTGAAAAC

2101 CTCTGACACA TGCAGCTCCC GGAGACGGTC ACAGCTTGTC TGTAAGCGGA TGCCGGGAGC

2161 AGACAAGCCC GTCAGGGCGC GTCAGCGGGT GTTGGCGGGT GTCGGGGCGG ATCGTCTTGC

2221 CTTGCTCGTC GGTGATGTAC TTCACCAGCT CCGCGAAGTC GCTCTTCTTG ATGGAGCGCA

2281 TGGGGACGTG CTTGGCAATC ACGCGCACCC CCCGGCCGTT TTAGCGGCTA AAAAAGTCAT

2341 GGCTCTGCCC TCGGGCGGAC CACGCCCATC ATGACCTTGC CAAGCTCGTC CTGCTTCTCT

2401 TCGATCTTCG CCAGCAGGGC GAGGATCGTG GCATCACCGA ACCGCGCCGT GCGCGGGTCG

2461 TCGGTGAGCC AGAGTTTCAG CAGGCCGCCC AGGCGGCCCA GGTCGCCATT GATGCGGGCC

2521 AGCTCGCGGA CGTGCTCATA GTCCACGACG CCCGTGATTT TGTAGCCCTG GCCGACGGCC

2581 AGCAGGTAGG CCGACAGGCT CATGCCGGCC GCCGCCGCCT TTTCCTCAAT CGCTCTTCGT

2641 TCGTCTGGAA GGCAGTACAC CTTGATAGGT GGGCTGCCCT TCCTGGTTGG CTTGGTTTCA

2701 TCAGCCATCC GCTTGCCCTC ATCTGTTACG CCGGCGGTAG CCGGCCAGCC TCGCAGAGCA

2761 GGATTCCCGT TGAGCACCGC CAGGTGCGAA TAAGGGACAG TGAAGAAGGA ACACCCGCTC

2821 GCGGGTGGGC CTACTTCACC TATCCTGCCC GGCTGACGCC GTTGGATACA CCAAGGAAAG

2881 TCTACACGAA CCCTTTGGCA AAATCCTGTA TATCGTGCGA AAAAGGATGG ATATACCGAA

2941 AAAATCGCTA TAATGACCCC GAAGCAGGGT TATGCAGCGG AAGATGCCAT TCGCCATTCA

3001 GGCTGCGCAA CTGTTGGGAA GGGCGATCGG TGCGGGCCTC TTCGCTATTA CGCCAGCTGG

3061 CGAAAGGGGG ATGTGCTGCA AGGCGATTAA GTTGGGTAAC GCCAGGGTTT TCCCAGTCAC

3121 GACGTTGTAA AACGACGGCC AGTGACCATG ATTACGCCAA GCTCGAAATT AACCCTCACT

3181 AAAGGGAACA AAAGCTGGTA CCTAACAGGA TTAGTGCAAT TCGAGTTGAA TCACTGGGAA

3241 AAACATTGTC TTCTTTTTTA TATTATCATT TGCATTAGTG CTGCAGTCGT AGATACTTGT

3301 TGGTTGAAAG ACATCAGCTG GGAGGGACTG GACTAGCGTT TGGTAAGGAG ACATACCTGT

3361 TAACGTTGGT TGCAAAATTC CATTTCGCGA TTTATGTTAT CTGTAAATCC TGATTTGTCT

3421 GGAATTCTTG ATACTTCCGT TTTTTTAGAG GCCAATGATT AGCATCGGCG ATTCTCAAAA

3481 TAGCATTTTC GACATGCGGT GCTGATTTCA TAAACATAGA CAACGCTTTT ACATGTAAAA

3541 GTAACTTGCG GACTTGGAAC AGTGCTCTGT TTTTGGTGTG AACGTAACTC AGCAATATTT

3601 CTGTGCTAGC AAGGTTTTTT ATGATCGACC GAAGATCTCA AAACTCCGGG TCTTTCAACT

3661 GTCTGACTAG ACCATGTTCG TAACGTCGGA CAGCAGCTTT CGTTGTACTG GTAGAATTTC

3721 TACGTGCGAA GCACGTGTAG GCAGGTTGAA CGACGATCCC TGCCGATGGA TGGATTGGCA

3781 CGCGGCGGAA CGCTTTCGTG ATCTACACCA CCTGGATCTT CACATATCTT CGAAATCGAA

3841 AAATTAACCA AGTCGACGGT ATCGATAATA TTCTAGCTGA GGGTACCCAT GGCCAAGTTG

3901 ACCAGTGCCG TTCCGGTGCT CACCGCGCGC GACGTCGCCG GAGCGGTCGA GTTCTGGACC

3961 GACCGGCTCG GGTTCTCCCG GGACTTCGTG GAGGACGACT TCGCCGGTGT GGTCCGGGAC

4021 GACGTGACCC TGTTCATCAG CGCGGTCCAG GACCAGGTGG TGCCGGACAA CACCCTGGCC

4081 TGGGTGTGGG TGCGCGGCCT GGACGAGCTG TACGCCGAGT GGTCGGAGGT CGTGTCCACG

4141 AACTTCCGGG ACGCCTCCGG GCCGGCCATG ACCGAGATCG GCGAGCAGCC GTGGGGGCGG

4201 GAGTTCGCCC TGCGCGACCC GGCCGGCAAC TGCGTGCACT TCGTGGCCGA GGAGCAGGAC

4261 TGACCGACGC CGACCAACAC CGCCGGTCCG ACGCGGCCCG ACGGGTCCGA GGCCTCGGAG

4321 ATCTGGGCCC ATGCGGCCGC AACAACTACC TCGACTTTGG CTGGGACACT TTCAGTGAGG

4381 ACAAGAAGCT TCAGAAGCGT GCTATCGAAC TCAACCAGGG ACGTGCGGCA CAAATGGGCA

4441 TCCTTGCTCT CATGGTGCAC GAACAGTTGG GAGTCTCTAT CCTTCCTTAA AAATTTAATT

4501 TTCATTAGTT GCAGTCACTC CGCTTTGGTT TCACAGTCAG GAATAACACT AGCTCGTCTT

4561 CACTCTAGAG TCGACCTGCA GGCATCTCTT CCGCTTCCTC GCTCACTGAC TCGCTGCGCT

4621 CGGTCGTTCG GCTGCGGCGA GCGGTATCAG CTCACTCAAA GGCGGTAATA CGGTTATCCA

4681 CAGAATCAGG GGATAACGCA GGAAAGAACA TGTGAGCAAA AGGCCAGCAA AAGGCCAGGA

4741 ACCGTAAAAA GGCCGCGTTG CTGGCGTTTT TCCATAGGCT CCGCCCCCCT GACGAGCATC

4801 ACAAAAATCG ACGCTCAAGT CAGAGGTGGC GAAACCCGAC AGGACTATAA AGATACCAGG

4861 CGTTTCCCCC TGGAAGCTCC CTCGTGCGCT CTCCTGTTCC GACCCTGCCG CTTACCGGAT

4921 ACCTGTCCGC CTTTCTCCCT TCGGGAAGCG TGGCGCTTTC TCATAGCTCA CGCTGTAGGT

4981 ATCTCAGTTC GGTGTAGGTC GTTCGCTCCA AGCTGGGCTG TGTGCACGAA CCCCCCGTTC

5041 AGCCCGACCG CTGCGCCTTA TCCGGTAACT ATCGTCTTGA GTCCAACCCG GTAAGACACG

5101 ACTTATCGCC ACTGGCAGCA GCCACTGGTA ACAGGATTAG CAGAGCGAGG TATGTAGGCG

5161 GTGCTACAGA GTTCTTGAAG TGGTGGCCTA ACTACGGCTA CACTAGAAGG ACAGTATTTG

5221 GTATCTGCGC TCTGCTGAAG CCAGTTACCT TCGGAAAAAG AGTTGGTAGC TCTTGATCCG

5281 GCAAACAAAC CACCGCTGGT AGCGGTGGTT TTTTTGTTTG CAAGCAGCAG ATTACGCGCA

5341 GAAAAAAAGG ATCTCAAGAA GATCCTTTGA TCTTTTCTAC GGGGTCTGAC GCTCAGTGGA

5401 ACGAAAACTC ACGTTAAGGG ATTTTGGTCA TGAGATTATC AAAAAGGATC TTCACCTAGA

5461 TCCTTTTAAA TTAAAAATGA AGTTTTAAAT CAATCTAAAG TATATATGAG TAAACTTGGT

5521 CTGACAGTTA CCAATGCTTA ATCAGTGAGG CACCTATCTC AGCGATCTGT CTATTTCGTT

5581 CATCCATAGT TGCCTGACTC CCCGTCGTGT AGATAACTAC GATACGGGAG GGCTTACCAT

5641 CTGGCCCCAG TGCTGCAATG ATACCGCGAG ACCCACGCTC ACCGGCTCCA GATTTATCAG

5701 CAATAAACCA GCCAGCCGGA AGGGCCGAGC GCAGAAGTGG TCCTGCAACT TTATCCGCCT

5761 CCATCCAGTC TATTAATTGT TGCCGGGAAG CTAGAGTAAG TAGTTCGCCA GTTAATAGTT

5821 TGCGCAACGT TGTTGCCATT GCTGCAGGCA TCGTGGTGTC ACGCTCGTCG TTTGGTATGG

5881 CTTCATTCAG CTCCGGTTCC CAACGATCAA GGCGAGTTAC ATGATCCCCC ATGTTGTGCA

5941 AAAAAGCGGT TAGCTCCTTC GGTCCTCCGA TCGTTGTCAG AAGTAAGTTG GCCGCAGTGT

6001 TATCACTCAT GGTTATGGCA GCACTGCATA ATTCTCTTAC TGTCATGCCA TCCGTAAGAT

6061 GCTTTTCTGT GACTGGTGAG TACTCAACCA AGTCATTCTG AGAATAGTGT ATGCGGCGAC

6121 CGAGTTGCTC TTGCCCGGCG TCAACACGGG ATAATACCGC GCCACATAGC AGAACTTTAA

6181 AAGTGCTCAT CATTGGAAAA CGTTCTTCGG GGCGAAAACT CTCAAGGATC TTACCGCTGT

6241 TGAGATCCAG TTCGATGTAA CCCACTCGTG CACCCAACTG ATCTTCAGCA TCTTTTACTT

6301 TCACCAGCGT TTCTGGGTGA GCAAAAACAG GAAGGCAAAA TGCCGCAAAA AAGGGAATAA

6361 GGGCGACACG GAAATGTTGA ATACTCATAC TCTTCCTTTT TCAATATTAT TGAAGCATTT

6421 ATCAGGGTTA TTGTCTCATG AGCGGATACA TATTTGAATG TATTTAGAAA AATAAACAAA

6481 TAGGGGTTCC GCGCACATTT CCCCGAAAAG TGCCACCTGA CGTCTAAGAA ACCATTATTA

6541 TCATGACATT AACCTATAAA AATAGGCGTA TCACGAGGCC CTTTCGTCTT CAAGAA

//

LOCUS pPtPBR11_sequenced 7300 bp DNA circular UNA 18-OCT-2007

DEFINITION Cloning vector pBR322, complete sequence.

KEYWORDS .

SOURCE Cloning vector pBR322 (unknown)

ORGANISM Cloning vector pBR322

other sequences; artificial sequences; vectors.

REFERENCE 1 (bases 1 to 4361)

AUTHORS New England Biolabs.

TITLE Direct Submission

JOURNAL Submitted (18-OCT-2007) Research Department, New England Biolabs,

240 County Road, Ipswich, MA 01938, USA

COMMENT 1. pBR322 beta-lactamase (bla, conferring ampicillin resistance)

is transcribed in vitro and in vivo both from its natural promoter

(P3) and from promoter P1, a pSC101 promoter artificially

juxtaposed with the bla sequence during construction of the pBR322

plasmid. (Ref: Brosius et al, J Biol Chem 257, 9205-9210 (1982)).

2. Corrects sequence error in tet gene deriving from the original

pSC101 sequence, present in GenBank entry (see accession J01749)

and NEB sequences dated 6/6/02 and earlier, based on sequencing of

pBR322 and pACYC184 at NEB in 2007 (Mathieu-Sheltry, V. and Cantin,

E., unpublished). Correction is C1134T, representing a Thr350Ile

correction in the expressed protein.

FEATURES Location/Qualifiers

source 1..7300

/organism="Cloning vector pBR322"

/mol_type="other DNA"

-35_signal 10..15

/note="tet (P2) promoter (clockwise); TTGACA"

-10_signal 33..38

/note="tet (P2) promoter (clockwise); TTTAAT; transcript

start 45"

-10_signal complement(44..49)

/note="P1 promoter (counter-clockwise); TAAACT"

-35_signal complement(64..75)

/note="P1 promoter -35 sequence region; transcript starts

36, 37 (complementary strand)"

CDS 86..1276

/gene="tet"

/note="tetR (confers resistance to tetracycline)"

/codon_start=1

/product="tetracycline efflux protein, class C"

/translation="MKSNNALIVILGTVTLDAVGIGLVMPVLPGLLRDIVHSDSIASH

YGVLLALYALMQFLCAPVLGALSDRFGRRPVLLASLLGATIDYAIMATTPVLWILYAG

RIVAGITGATGAVAGAYIADITDGEDRARHFGLMSACFGVGMVAGPVAGGLLGAISLH

APFLAAAVLNGLNLLLGCFLMQESHKGERRPMPLRAFNPVSSFRWARGMTIVAALMTV

FFIMQLVGQVPAALWVIFGEDRFRWSATMIGLSLAVFGILHALAQAFVTGPATKRFGE

KQAIIAGMAADALGYVLLAFATRGWMAFPIMILLASGGIGMPALQAMLSRQVDDDHQG

QLQGSLAALTSLTSIIGPLIVTAIYAASASTWNGLAWIVGAALYLVCLPALRRGAWSR

ATST"

gene 86..1276

/gene="tet"

RBS 1905..1909

/note="rop RBS"

CDS 1915..2106

/gene="rop"

/exception="alternative start codon"

/codon_start=1

/transl_table=11

/product="ROP protein"

/translation="MTKQEKTALNMARFIRSQTLTLLEKLNELDADEQADICESLHDH

ADELYRSCLARFGDDGENL"

gene 1915..2106

/gene="rop"

primer 2169..2239

/label=Insert-F

primer complement(2191..2209)

/label=pBR322-R

OriT 2210..2985

/label=OriT-pRL2948a

Insert 2210..5289

ShBle 3145..4562

/label=PtBle

CEN 4563..4679

Ars 4680..5067

HIS 5068..5266

primer complement(5271..5330)

/label=Insert-R

primer 5290..5308

/label=pBR322-F

misc_RNA complement(5473..6025)

/note="RNAII transcript (complementary strand)"

rep_origin complement(5473..6061)

/note="pMB1 origin of replication (counter-clockwise)

(RNAII -35 to RNA/DNA switch point)"

-35_signal 5879..5884

/note="RNAI promoter (clockwise); TTGAAG"

-10_signal 5901..5906

/note="RNAI promoter (clockwise); GCTACA"

misc_RNA 5915..6022

/note="RNAI transcript"

-10_signal complement(6035..6040)

/note="RNAII promoter (counter-clockwise); CGTAAT"

-35_signal complement(6056..6061)

/note="RNAII promoter (counter-clockwise); TTGAGA"

primer 6101..6123

/label=Amp-Primer-R

CDS complement(6232..7092)

/gene="bla"

/note="ampR (confers resistance to ampicillin)"

/codon_start=1

/product="beta-lactamase"

/translation="MSIQHFRVALIPFFAAFCLPVFAHPETLVKVKDAEDQLGARVGY

IELDLNSGKILESFRPEERFPMMSTFKVLLCGAVLSRVDAGQEQLGRRIHYSQNDLVE

YSPVTEKHLTDGMTVRELCSAAITMSDNTAANLLLTTIGGPKELTAFLHNMGDHVTRL

DRWEPELNEAIPNDERDTTMPAAMATTLRKLLTGELLTLASRQQLIDWMEADKVAGPL

LRSALPAGWFIADKSGAGERGSRGIIAALGPDGKPSRIVVIYTTGSQATMDERNRQIA

EIGASLIKHW"

gene complement(6232..7092)

/gene="bla"

sig_peptide complement(7024..7092)

/gene="bla"

/note="required for secretion to the periplasm; cleaved

off to form the mature beta-lactamase protein."

RBS complement(7100..7104)

/note="bla RBS"

-10_signal complement(7136..7141)

/note="bla (P3) promoter (counter-clockwise); GAGACA;

transcript start 4188 (complementary strand)"

-35_signal complement(7157..7162)

/note="bla (P3) promoter (counter-clockwise); TTCAAA"

primer complement(7279..7298)

/label=Amp-Primer-F

ORIGIN

1 TTCTCATGTT TGACAGCTTA TCATCGATAA GCTTTAATGC GGTAGTTTAT CACAGTTAAA

61 TTGCTAACGC AGTCAGGCAC CGTGTATGAA ATCTAACAAT GCGCTCATCG TCATCCTCGG

121 CACCGTCACC CTGGATGCTG TAGGCATAGG CTTGGTTATG CCGGTACTGC CGGGCCTCTT

181 GCGGGATATC GTCCATTCCG ACAGCATCGC CAGTCACTAT GGCGTGCTGC TAGCGCTATA

241 TGCGTTGATG CAATTTCTAT GCGCACCCGT TCTCGGAGCA CTGTCCGACC GCTTTGGCCG

301 CCGCCCAGTC CTGCTCGCTT CGCTACTTGG AGCCACTATC GACTACGCGA TCATGGCGAC

361 CACACCCGTC CTGTGGATCC TCTACGCCGG ACGCATCGTG GCCGGCATCA CCGGCGCCAC

421 AGGTGCGGTT GCTGGCGCCT ATATCGCCGA CATCACCGAT GGGGAAGATC GGGCTCGCCA

481 CTTCGGGCTC ATGAGCGCTT GTTTCGGCGT GGGTATGGTG GCAGGCCCCG TGGCCGGGGG

541 ACTGTTGGGC GCCATCTCCT TGCATGCACC ATTCCTTGCG GCGGCGGTGC TCAACGGCCT

601 CAACCTACTA CTGGGCTGCT TCCTAATGCA GGAGTCGCAT AAGGGAGAGC GTCGACCGAT

661 GCCCTTGAGA GCCTTCAACC CAGTCAGCTC CTTCCGGTGG GCGCGGGGCA TGACTATCGT

721 CGCCGCACTT ATGACTGTCT TCTTTATCAT GCAACTCGTA GGACAGGTGC CGGCAGCGCT

781 CTGGGTCATT TTCGGCGAGG ACCGCTTTCG CTGGAGCGCG ACGATGATCG GCCTGTCGCT

841 TGCGGTATTC GGAATCTTGC ACGCCCTCGC TCAAGCCTTC GTCACTGGTC CCGCCACCAA

901 ACGTTTCGGC GAGAAGCAGG CCATTATCGC CGGCATGGCG GCCGACGCGC TGGGCTACGT

961 CTTGCTGGCG TTCGCGACGC GAGGCTGGAT GGCCTTCCCC ATTATGATTC TTCTCGCTTC

1021 CGGCGGCATC GGGATGCCCG CGTTGCAGGC CATGCTGTCC AGGCAGGTAG ATGACGACCA

1081 TCAGGGACAG CTTCAAGGAT CGCTCGCGGC TCTTACCAGC CTAACTTCGA TCATTGGACC

1141 GCTGATCGTC ACGGCGATTT ATGCCGCCTC GGCGAGCACA TGGAACGGGT TGGCATGGAT

1201 TGTAGGCGCC GCCCTATACC TTGTCTGCCT CCCCGCGTTG CGTCGCGGTG CATGGAGCCG

1261 GGCCACCTCG ACCTGAATGG AAGCCGGCGG CACCTCGCTA ACGGATTCAC CACTCCAAGA

1321 ATTGGAGCCA ATCAATTCTT GCGGAGAACT GTGAATGCGC AAACCAACCC TTGGCAGAAC

1381 ATATCCATCG CGTCCGCCAT CTCCAGCAGC CGCACGCGGC GCATCTCGGG CAGCGTTGGG

1441 TCCTGGCCAC GGGTGCGCAT GATCGTGCTC CTGTCGTTGA GGACCCGGCT AGGCTGGCGG

1501 GGTTGCCTTA CTGGTTAGCA GAATGAATCA CCGATACGCG AGCGAACGTG AAGCGACTGC

1561 TGCTGCAAAA CGTCTGCGAC CTGAGCAACA ACATGAATGG TCTTCGGTTT CCGTGTTTCG

1621 TAAAGTCTGG AAACGCGGAA GTCAGCGCCC TGCACCATTA TGTTCCGGAT CTGCATCGCA

1681 GGATGCTGCT GGCTACCCTG TGGAACACCT ACATCTGTAT TAACGAAGCG CTGGCATTGA

1741 CCCTGAGTGA TTTTTCTCTG GTCCCGCCGC ATCCATACCG CCAGTTGTTT ACCCTCACAA

1801 CGTTCCAGTA ACCGGGCATG TTCATCATCA GTAACCCGTA TCGTGAGCAT CCTCTCTCGT

1861 TTCATCGGTA TCATTACCCC CATGAACAGA AATCCCCCTT ACACGGAGGC ATCAGTGACC

1921 AAACAGGAAA AAACCGCCCT TAACATGGCC CGCTTTATCA GAAGCCAGAC ATTAACGCTT

1981 CTGGAGAAAC TCAACGAGCT GGACGCGGAT GAACAGGCAG ACATCTGTGA ATCGCTTCAC

2041 GACCACGCTG ATGAGCTTTA CCGCAGCTGC CTCGCGCGTT TCGGTGATGA CGGTGAAAAC

2101 CTCTGACACA TGCAGCTCCC GGAGACGGTC ACAGCTTGTC TGTAAGCGGA TGCCGGGAGC

2161 AGACAAGCCC GTCAGGGCGC GTCAGCGGGT GTTGGCGGGT GTCGGGGCGG ATCGTCTTGC

2221 CTTGCTCGTC GGTGATGTAC TTCACCAGCT CCGCGAAGTC GCTCTTCTTG ATGGAGCGCA

2281 TGGGGACGTG CTTGGCAATC ACGCGCACCC CCCGGCCGTT TTAGCGGCTA AAAAAGTCAT

2341 GGCTCTGCCC TCGGGCGGAC CACGCCCATC ATGACCTTGC CAAGCTCGTC CTGCTTCTCT

2401 TCGATCTTCG CCAGCAGGGC GAGGATCGTG GCATCACCGA ACCGCGCCGT GCGCGGGTCG

2461 TCGGTGAGCC AGAGTTTCAG CAGGCCGCCC AGGCGGCCCA GGTCGCCATT GATGCGGGCC

2521 AGCTCGCGGA CGTGCTCATA GTCCACGACG CCCGTGATTT TGTAGCCCTG GCCGACGGCC

2581 AGCAGGTAGG CCGACAGGCT CATGCCGGCC GCCGCCGCCT TTTCCTCAAT CGCTCTTCGT

2641 TCGTCTGGAA GGCAGTACAC CTTGATAGGT GGGCTGCCCT TCCTGGTTGG CTTGGTTTCA

2701 TCAGCCATCC GCTTGCCCTC ATCTGTTACG CCGGCGGTAG CCGGCCAGCC TCGCAGAGCA

2761 GGATTCCCGT TGAGCACCGC CAGGTGCGAA TAAGGGACAG TGAAGAAGGA ACACCCGCTC

2821 GCGGGTGGGC CTACTTCACC TATCCTGCCC GGCTGACGCC GTTGGATACA CCAAGGAAAG

2881 TCTACACGAA CCCTTTGGCA AAATCCTGTA TATCGTGCGA AAAAGGATGG ATATACCGAA

2941 AAAATCGCTA TAATGACCCC GAAGCAGGGT TATGCAGCGG AAGATGCCAT TCGCCATTCA

3001 GGCTGCGCAA CTGTTGGGAA GGGCGATCGG TGCGGGCCTC TTCGCTATTA CGCCAGCTGG

3061 CGAAAGGGGG ATGTGCTGCA AGGCGATTAA GTTGGGTAAC GCCAGGGTTT TCCCAGTCAC

3121 GACGTTGTAA AACGACGGCC AGTGACCATG ATTACGCCAA GCTCGAAATT AACCCTCACT

3181 AAAGGGAACA AAAGCTGGTA CCTAACAGGA TTAGTGCAAT TCGAGTTGAA TCACTGGGAA

3241 AAACATTGTC TTCTTTTTTA TATTATCATT TGCATTAGTG CTGCAGTCGT AGATACTTGT

3301 TGGTTGAAAG ACATCAGCTG GGAGGGACTG GACTAGCGTT TGGTAAGGAG ACATACCTGT

3361 TAACGTTGGT TGCAAAATTC CATTTCGCGA TTTATGTTAT CTGTAAATCC TGATTTGTCT

3421 GGAATTCTTG ATACTTCCGT TTTTTTAGAG GCCAATGATT AGCATCGGCG ATTCTCAAAA

3481 TAGCATTTTC GACATGCGGT GCTGATTTCA TAAACATAGA CAACGCTTTT ACATGTAAAA

3541 GTAACTTGCG GACTTGGAAC AGTGCTCTGT TTTTGGTGTG AACGTAACTC AGCAATATTT

3601 CTGTGCTAGC AAGGTTTTTT ATGATCGACC GAAGATCTCA AAACTCCGGG TCTTTCAACT

3661 GTCTGACTAG ACCATGTTCG TAACGTCGGA CAGCAGCTTT CGTTGTACTG GTAGAATTTC

3721 TACGTGCGAA GCACGTGTAG GCAGGTTGAA CGACGATCCC TGCCGATGGA TGGATTGGCA

3781 CGCGGCGGAA CGCTTTCGTG ATCTACACCA CCTGGATCTT CACATATCTT CGAAATCGAA

3841 AAATTAACCA AGTCGACGGT ATCGATAATA TTCTAGCTGA GGGTACCCAT GGCCAAGTTG

3901 ACCAGTGCCG TTCCGGTGCT CACCGCGCGC GACGTCGCCG GAGCGGTCGA GTTCTGGACC

3961 GACCGGCTCG GGTTCTCCCG GGACTTCGTG GAGGACGACT TCGCCGGTGT GGTCCGGGAC

4021 GACGTGACCC TGTTCATCAG CGCGGTCCAG GACCAGGTGG TGCCGGACAA CACCCTGGCC

4081 TGGGTGTGGG TGCGCGGCCT GGACGAGCTG TACGCCGAGT GGTCGGAGGT CGTGTCCACG

4141 AACTTCCGGG ACGCCTCCGG GCCGGCCATG ACCGAGATCG GCGAGCAGCC GTGGGGGCGG

4201 GAGTTCGCCC TGCGCGACCC GGCCGGCAAC TGCGTGCACT TCGTGGCCGA GGAGCAGGAC

4261 TGACCGACGC CGACCAACAC CGCCGGTCCG ACGCGGCCCG ACGGGTCCGA GGCCTCGGAG

4321 ATCTGGGCCC ATGCGGCCGC AACAACTACC TCGACTTTGG CTGGGACACT TTCAGTGAGG

4381 ACAAGAAGCT TCAGAAGCGT GCTATCGAAC TCAACCAGGG ACGTGCGGCA CAAATGGGCA

4441 TCCTTGCTCT CATGGTGCAC GAACAGTTGG GAGTCTCTAT CCTTCCTTAA AAATTTAATT

4501 TTCATTAGTT GCAGTCACTC CGCTTTGGTT TCACAGTCAG GAATAACACT AGCTCGTCTT

4561 CAATCACGTG CTATAAAAAT AATTATAATT TAAATTTTTT AATATAAATA TATAAATTAA

4621 AAATAGAAAG TAAAAAAAGA AATTAAAGAA AAAATAGTTT TTGTTTTCCG AAGATGTAAA

4681 AGACTCTAGG GGGATCGCCA ACAAATACTA CCTTTTACCT TGCTCTTCCT GCTCTCAGGT

4741 ATTAATGCCG AATTGTTTCA TCTTGTCTGT GTAGAAGACC ACACACGAAA ATCCTGTGAT

4801 TTTACATTTT ACTTATCGTT AATCGAATGT ATATCTATTT AATCTGCTTT TCTTGTCTAA

4861 TAAATATATA TGTAAAGTAC GCTTTTTGTT GAAATTTTTT AAACCTTTGT TTATTTTTTT

4921 TTCTTCATTC CGTAACTCTT CTACCTTCTT TATTTACTTT CTAAAATCCA AATACAAAAC

4981 ATAAAAATAA ATAAACACAG AGTAAATTCC CAAATTATTC CATCATTAAA AGATACGAGG

5041 CGCGTGTAAG TTACAGGCAA GCGATCCTAG TACACTCTAT ATTTTTTTAT GCCTCGGTAA

5101 TGATTTTCAT TTTTTTTTTC CACCTAGCGG ATGACTCTTT TTTTTTCTTA GCGATTGGCA

5161 TTATCACATA ATGAATTATA CATTATATAA AGTAATGTGA TTTCTTCGAA GAATATACTA

5221 AAAAATGAGC AGGCAAGATA AACGAAGGCA AAGATGACAG AGCAGACTCT AGAGTCGACC

5281 TGCAGGCATC TCTTCCGCTT CCTCGCTCAC TGACTCGCTG CGCTCGGTCG TTCGGCTGCG

5341 GCGAGCGGTA TCAGCTCACT CAAAGGCGGT AATACGGTTA TCCACAGAAT CAGGGGATAA

5401 CGCAGGAAAG AACATGTGAG CAAAAGGCCA GCAAAAGGCC AGGAACCGTA AAAAGGCCGC

5461 GTTGCTGGCG TTTTTCCATA GGCTCCGCCC CCCTGACGAG CATCACAAAA ATCGACGCTC

5521 AAGTCAGAGG TGGCGAAACC CGACAGGACT ATAAAGATAC CAGGCGTTTC CCCCTGGAAG

5581 CTCCCTCGTG CGCTCTCCTG TTCCGACCCT GCCGCTTACC GGATACCTGT CCGCCTTTCT

5641 CCCTTCGGGA AGCGTGGCGC TTTCTCATAG CTCACGCTGT AGGTATCTCA GTTCGGTGTA

5701 GGTCGTTCGC TCCAAGCTGG GCTGTGTGCA CGAACCCCCC GTTCAGCCCG ACCGCTGCGC

5761 CTTATCCGGT AACTATCGTC TTGAGTCCAA CCCGGTAAGA CACGACTTAT CGCCACTGGC

5821 AGCAGCCACT GGTAACAGGA TTAGCAGAGC GAGGTATGTA GGCGGTGCTA CAGAGTTCTT

5881 GAAGTGGTGG CCTAACTACG GCTACACTAG AAGGACAGTA TTTGGTATCT GCGCTCTGCT

5941 GAAGCCAGTT ACCTTCGGAA AAAGAGTTGG TAGCTCTTGA TCCGGCAAAC AAACCACCGC

6001 TGGTAGCGGT GGTTTTTTTG TTTGCAAGCA GCAGATTACG CGCAGAAAAA AAGGATCTCA

6061 AGAAGATCCT TTGATCTTTT CTACGGGGTC TGACGCTCAG TGGAACGAAA ACTCACGTTA

6121 AGGGATTTTG GTCATGAGAT TATCAAAAAG GATCTTCACC TAGATCCTTT TAAATTAAAA

6181 ATGAAGTTTT AAATCAATCT AAAGTATATA TGAGTAAACT TGGTCTGACA GTTACCAATG

6241 CTTAATCAGT GAGGCACCTA TCTCAGCGAT CTGTCTATTT CGTTCATCCA TAGTTGCCTG

6301 ACTCCCCGTC GTGTAGATAA CTACGATACG GGAGGGCTTA CCATCTGGCC CCAGTGCTGC

6361 AATGATACCG CGAGACCCAC GCTCACCGGC TCCAGATTTA TCAGCAATAA ACCAGCCAGC

6421 CGGAAGGGCC GAGCGCAGAA GTGGTCCTGC AACTTTATCC GCCTCCATCC AGTCTATTAA

6481 TTGTTGCCGG GAAGCTAGAG TAAGTAGTTC GCCAGTTAAT AGTTTGCGCA ACGTTGTTGC

6541 CATTGCTGCA GGCATCGTGG TGTCACGCTC GTCGTTTGGT ATGGCTTCAT TCAGCTCCGG

6601 TTCCCAACGA TCAAGGCGAG TTACATGATC CCCCATGTTG TGCAAAAAAG CGGTTAGCTC

6661 CTTCGGTCCT CCGATCGTTG TCAGAAGTAA GTTGGCCGCA GTGTTATCAC TCATGGTTAT

6721 GGCAGCACTG CATAATTCTC TTACTGTCAT GCCATCCGTA AGATGCTTTT CTGTGACTGG

6781 TGAGTACTCA ACCAAGTCAT TCTGAGAATA GTGTATGCGG CGACCGAGTT GCTCTTGCCC

6841 GGCGTCAACA CGGGATAATA CCGCGCCACA TAGCAGAACT TTAAAAGTGC TCATCATTGG

6901 AAAACGTTCT TCGGGGCGAA AACTCTCAAG GATCTTACCG CTGTTGAGAT CCAGTTCGAT

6961 GTAACCCACT CGTGCACCCA ACTGATCTTC AGCATCTTTT ACTTTCACCA GCGTTTCTGG

7021 GTGAGCAAAA ACAGGAAGGC AAAATGCCGC AAAAAAGGGA ATAAGGGCGA CACGGAAATG

7081 TTGAATACTC ATACTCTTCC TTTTTCAATA TTATTGAAGC ATTTATCAGG GTTATTGTCT

7141 CATGAGCGGA TACATATTTG AATGTATTTA GAAAAATAAA CAAATAGGGG TTCCGCGCAC

7201 ATTTCCCCGA AAAGTGCCAC CTGACGTCTA AGAAACCATT ATTATCATGA CATTAACCTA

7261 TAAAAATAGG CGTATCACGA GGCCCTTTCG TCTTCAAGAA

//
