# Supplementary material for: Refinement of the Diatom Episome Maintenance Sequence and Improvement of Conjugation-Based DNA Delivery Methods
Source: Front Bioeng Biotechnol. 2016 Aug 8;4:65. doi: 10.3389/fbioe.2016.00065 (PMC4976089; doi:10.3389/fbioe.2016.00065)
Supplement: Supplementary file 8 [file Image_1.PDF]

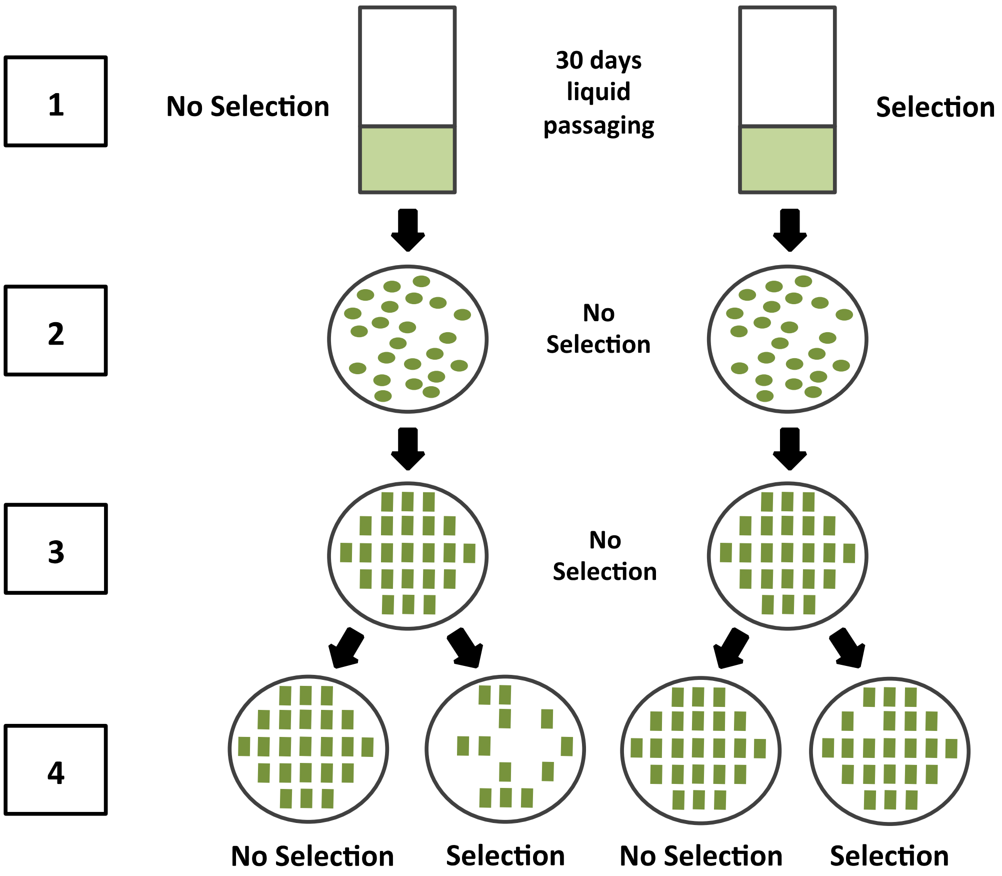

Construct	Clone	% maintained with selection	% maintained without selection
**pPtPBR1**	1	99	53
	8	90	43
**pPtPBR6**	9	89	31
**pPtPBR8**	4	87	5
	1	97	67

Supplementary Figure 1. Passaging experiment to determine whether contructs containing the entire *CEN6-ARSH4-HIS3* sequence (pPTPBR1), the *CEN6-ARSH4* sequence only (pPTPBR6), and 2 copies of the *CEN6-ARSH4* sequence on different parts of the episome (pPTPBR8) can be maintained in *P. tricornutum* with and without selection. Figure A depicts the experimental design in 4 steps: 1) passaging liquid semi-continuous cultures of ex-conjugants with and without selection for 30 days, 2) plating diluted cultures without selection, 3) selecting colonies and patching on plates without selection, and 4) patching each colony on plates with and without selection. Figure B reports the percentage of ex-conjugants that maintained antibiotic resistance after passaging with and without selection for each construct.

A= Top Figure
B = Table beneath
